# *Lactobacillus casei* Shirota probiotic drinks reduce antibiotic associated diarrhoea in patients with spinal cord injuries who regularly consume proton pump inhibitors: a subgroup analysis of the ECLISP multicentre RCT

**DOI:** 10.1038/s41393-024-00983-w

**Published:** 2024-03-22

**Authors:** Samford Wong, Shashivadan P. Hirani, Alastair Forbes, Naveen Kumar, Ramaswamy Hariharan, Jean O’Driscoll, Ravi Sekhar, Ali Jamous

**Affiliations:** 1 National Spinal Injuries Centre, Stoke Mandeville Hospital, Aylesbury, UK; 2 School of Health & Psychological Sciences, City, University of London, London, UK; 3 Royal Buckinghamshire Hospital, Aylesbury, UK; 4 University of Tartu, Estonia, and Norwich Medical School, University of East Anglia, Norwich, UK; 5 Midland Centre for Spinal Injury, Robert Jones and Agnes Hunt Orthopaedic Hospital, Gobowen, UK; 6 The Princess Royal Spinal Injuries Centre, Northern General Hospital, Sheffield, UK; 7 Department of Microbiology, Stoke Mandeville Hospital, Aylesbury, UK; 8 Department of Gastroenterology, Stoke Mandeville Hospital, Aylesbury, UK; 9 INRES Neuro, Wendover, UK

**Keywords:** Clostridium difficile, Nutrition, Nutritional supplements

## Abstract

**Study design:**

This was a sub-group analysis of a multicentre, randomised, placebo-controlled, double-blind trial (ECLISP trial)

**Objectives:**

To assess the efficacy of a probiotic containing at least 6.5 × 10^9^ live *Lactobacillus casei* Shirota (LcS) in preventing antibiotic associated diarrhoea (AAD) in patients with spinal cord injury (SCI) who consumed proton pump inhibitor (PPI) regularly. LcS or placebo was given once daily for the duration of an antibiotic course and continued for 7 days thereafter. The trial was registered with ISRCTN:13119162.

**Setting:**

Three SCI centres (National Spinal Injuries Centre, Midland Centre for Spinal Injuries and Princess Royal Spinal Cord Injuries Centre) in the United Kingdom

**Methods:**

Between November 2014, and November 2019, 95 eligible consenting SCI patients (median age: 57; IQ range: 43-69) were randomly allocated to receive LcS (*n* = 50) or placebo (*n* = 45). The primary outcome is the occurrence of AAD up to 30 days after finishing LcS/placebo.

**Results:**

The LcS group had a significantly lower incidence of AAD at 30 days after finishing the antibiotic course (28.0 v 53.3%, RR: 95% CI: 0.53, 0.31–0.89; z = 2.5, *p* = 0.01). Multivariate logistic regression analysis identified that LcS can reduce the risk of AAD at 30 days (OR: 0.36, 95% CI 0.13, 0.99, *p* < 0.05). No intervention-related adverse events were reported during the study.

**Conclusions:**

LcS has the potential to prevent AAD in what could be considered a defined vulnerable group of SCI patients on regular PPI. A confirmatory, randomised, placebo-controlled study is needed to confirm this apparent therapeutic success to translate it into appropriate clinical outcomes.

**Sponsorship:**

Yakult Honsha Co., Ltd.

## Introduction

Spinal cord injury (SCI) is a catastrophic condition that affects at least 2,500 people in the UK annually [1]. Neurogenic bladder dysfunction because of SCI often leads to increased risk of symptomatic urinary tract infections [2, 3]. The use of urinary catheters further increases the need for antibiotics and the risk of the undesirable effects like antibiotic associated diarrhoea (AAD) and *Clostridioides difficile* infection (CDI) [4]. In addition, diarrhoea can moreover delay rehabilitation, increase the risk of developing pressure ulcers/delay wound healing and reduce quality of life.

During the acute stage, people with SCI (PWSCI) require anticoagulation therapy to prevent venous thromboembolism. This and the increased risk of upper gastrointestinal haemorrhage due to spinal cord damage, patients are prescribed gastric protection, such as proton pump inhibitor (PPI). However, PPI exposure is also a risk factor for AAD/CDI. Literature reports show that patients on PPIs have a relative risk of 1.69 of contracting CDI compared to patients who are not taking the medication [5]. The prevalence of AAD and CDI in PWSCI are reported in the range 14.9–30.3% [6–8].

Our previous RCT [9], using a strict criteria for defining AAD (≥2 liquid stools using Bristol Stool Scale type 5, 6 or 7) over a 24 hours period, indicated that probiotic, *Lactobacillus casei* Shirota (LcS), may have a potential to prevent AAD in the subgroup of PWSCI on PPI.

There is growing interest in probiotics to reduce the risk of AAD/CDI in general. Probiotics, defined as ‘*live microorganisms that, when administered in adequate amounts, confer a health benefit on the host’*, have been proposed to prevent AAD/CDI by restoring or maintaining a healthy gut microbiome in hospitalised patients on antibiotic therapy, particularly those on broad-spectrum antibiotics [10]. However, it is still unclear whether a specific probiotic strains is responsible to reduce the overall incidence of AAD/CDI. In order to confirms this effect, we carried out a sub-group analysis to assess the efficacy of live LcS in preventing AAD in people with SCI who are on PPI regularly.

## Methods

### Study design

This was a sub-group analysis from a prospective, multicentre, randomised, double-blind, placebo-controlled study (ECLISP) [9]. Patients who had been prescribed antibiotics were identified and approached for consent. After obtaining written informed consent, study data were collected at the time of prescribing antibiotics (baseline) and at follow up, set at 7 days and 30 days after the end of the antibiotic course (Abx + 7d, Abx + 30d). The study was conducted within the National Health Service in the UK. The three centres involved in this study are responsible for about 45–50% of all specialist SCI service in the UK.

### Participants

The inclusion criteria of the present sub-group analysis included patients aged ≥18 years, who had sustained a SCI, had been admitted to one of the three investigatory centres, were due to receive antibiotics for an infection, who are taking PPI regularly and who were able to take the study drinks within 48 h of the first dose of antibiotic. Patients were excluded from the study for the following reasons: patients could not be recruited more than once and if they had antibiotic use in the 30 days prior to recruitment – although a single dose of prophylactic antibiotic given 14 to 30 days before recruitment was permitted. Also, patients with diarrhoea within the seven days prior to recruitment, and those with known gastrointestinal disease that could result in diarrhoea as well as patients with several other conditions and comorbidities, were excluded. (Supplementary Table: Appendix 1).

Between November 2014 to November 2019, 95 consenting SCI patients, who were within 48 h of commencing antibiotics and taking regular PPI, were randomly allocated to receive a fermented milk drink (Yakult^®^: 65 ml) containing a minimum of 6.5 ×10^9^ colony-forming units (CFU) LcS/bottle, or placebo daily for the duration of the antibiotic course and for 7 days thereafter. The study drink was given at the drug round by nurses. Consumption was monitored on a daily basis by the study team. Minor non-compliance was defined as two consecutive days of not drinking the study intervention. Major non-compliance was defined when three or more consecutive days were missed. If participants missed the intervention for more than three days, they were withdrawn from the study.

The participants’ demographics, baseline clinical and nutritional information were collected. These included age, gender, level of SCI and completeness of injury using the International Standards for Neurological Classification of Spinal Cord Injury [11] and the cause of SCI. Information about nutritional factors, such as weight and height, route of nutrition, nutrient intake as estimated by food record charts (nil by mouth, less than half, half, more than half, and all eaten), interruptions and supplementation of nutrition (use of oral nutritional supplements and artificial nutrition support), were collected. Additional data, which included the use of mechanical ventilation, the history of intensive care unit stay, the number of medications, the indication, route and the antibiotic used as well as the use of laxatives, were recorded.

The perceived risks of the various antibiotics were used to categorise patients into three groups: “low-risk” antibiotics (metronidazole and parenteral aminoglycosides), “medium risk” antibiotics (tetracyclines, sulphonamides and macrolides) and “high risk” antibiotics (aminopenicillins, cephalosporins and quinolones) as described in previous studies [9, 12] and by the UK National Institute for Health and Care Excellence [10].

### Primary outcome

The primary outcome was defined as the occurrence of AAD during and up to 30 days after the antibiotic course finished. The bowel movements were monitored routinely by the nursing staff on the ward using the Bristol stool scale [13]. Diarrhoea was defined as more than two liquid stools (Bristol stool scale type 5, 6 or 7) in any 24 h period.

### Secondary outcomes

Whenever diarrhoea was reported, a stool sample was collected and sent to the hospital laboratory for the detection of *C. difficile* toxin. In the present study, CDI was defined by the hospital microbiology laboratory on confirmation of the presence of *C. difficile* toxin, but the method of *C. difficile* toxin detection varied between the laboratories: i.e., screening for glutamate dehydrogenase (GDH) antigen followed by toxin A and B detection, enzyme immunoassays for *C. difficile* toxin A and toxin B, or toxin-producing *C. difficile* gene detection by polymerase chain reaction testing, or a combination of these [14]. The study team recorded the occurrence of diarrhoea throughout the study. The census date was fixed 30 days after the antibiotic course had finished. The secondary outcomes were the occurrence of AAD during and up to 7 days after the antibiotic course finished with or without CDI being detected by the study site laboratory.

### Statistical analysis

The primary statistical analysis was carried out on the basis of intention-to-treat, with all participants being analysed according to their allocated treatment group irrespective of what treatment they actually received.

Fisher’s exact test and χ^2^ test were used to compare rates of diarrhoea, as well as rates of AAD and CDI across categorical variables. Relative risks with 95% confidence intervals, were used to describe the treatment effects of LcS.

A series of screening univariate analyses were undertaken. Logistic regressions were used to establish which factors individually influenced the occurrence of diarrhoea and its duration as well as the development of CDI at Abx + 7d and Abx + 30d follow up. Linear regression was used for continuous outcome measures for the duration of diarrhoea and the number of episodes of diarrhoea. Thereafter, statistically significant univariate predictors were used in, multiple binary logistic regression and multiple regression analysis to determine statistically significant predictors for AAD, CDI and other secondary outcomes, after accounting for their relationship to other pertinent variables. No allowance for multiplicity was made for the secondary outcomes.

To reduce the bias implicit in utilising only complete cases, multiple imputation procedures for the data was used using the SPSS (SPSS version 25, Inc, Chicago, IL) multiple imputation function with fully conditional specification (maximum iterations of 500) using a Predictive Mean Modelling method (PMM) to produce 10 imputed datasets. The imputation model included all variables (demographic, clinical and outcomes) involved in the analyses, with PMM imputed variable limits set for imputed values to be within the range of available data. Main outcome variables i.e., AAD and CDI were not imputed. These 10 imputed datasets were then individually analysed as normal; thereafter standard multiple imputation procedures were used to combine the multiple scalar and multivariate estimates quantities [15, 16]. This reduces the bias of analysing incomplete datasets.

For logistic regression, odds ratio (OR), Nagelkerke’s (R) and correctly classified cases are reported. For linear regression, adjusted R^2^ and β coefficients with t-test significance are reported. For all tests, a *p*-value of 0.05 or less, or when the 95% CI for OR did not cross 1.0 were considered as statistically significant. Statistical analysis was performed using the Minitab statistical software (Version 25.0; Minitab, Inc.) and SPSS (Version 19; IBM Corporations).

### Ethical consideration

The present study, conducted according to the guidelines laid down in the Declaration of Helsinki, received ethical approval from the National Research Ethics (REC) Committee (reference no. 14/SC/1101) and approval from the local research and development department at each participating site. After the study had been explained by a research coordinator and all questions had been answered, each participant signed an informed consent form prior to trial initiation.

The original study protocol was registered at ISRCTN in January 2015 (ISRCTN13119162). The study steering group was set up in July 2014 and the study commenced its recruitment in November 2014. Changes were recommended to the original, approved protocol to improve recruitment specifically in relation to the inclusion and exclusion criteria of patients’ enrolment. The new version of the protocol was developed in accordance with the consolidated standards of reporting trials 2010 guideline and subsequently approved by the sponsor and funder and the REC [Supplementary File].

A register was kept of all patients who withdrew from the study. The reason for consent withdrawal was documented, if provided by the participants, along with the following core patient characteristics: age, gender and level and severity of SCI. To monitor the progress and conduct of the study, all investigators attended meetings before the study and met for communal bi-annual updates and end of study meeting in Jan 2019.

The study was additionally monitored by an external Clinical Research Associate (PHARMExcel) according to applicable provisions of the sponsor’s subcontractors monitoring procedures, in conformance with ICH-GCP FDA guidelines, ISO 14155 and UK-specific laws/regulations.

### Definition of undernutrition risk

Participants were considered at risk of undernutrition on the basis of the Spinal Nutrition Screening Tool (SNST) [17]. The SNST assesses eight criteria, of which the majority are recognised as predictors or symptoms of undernutrition: history of recent weight loss; body mass index; level of SCI; presence of co-morbidity; skin condition; appetite; ability to eat. Each step of screening has a score of up to 5 and the total score reflects the participant’s degree of risk. A score of 0–10 suggests a low risk, 11–15 a moderate risk and >15 suggests a high risk of undernutrition. Participants who had a SNST score ≤10 were considered at low risk, and all those with a SNST score ≥11 were considered at increased risk.

## Results

**Fig. 1:**
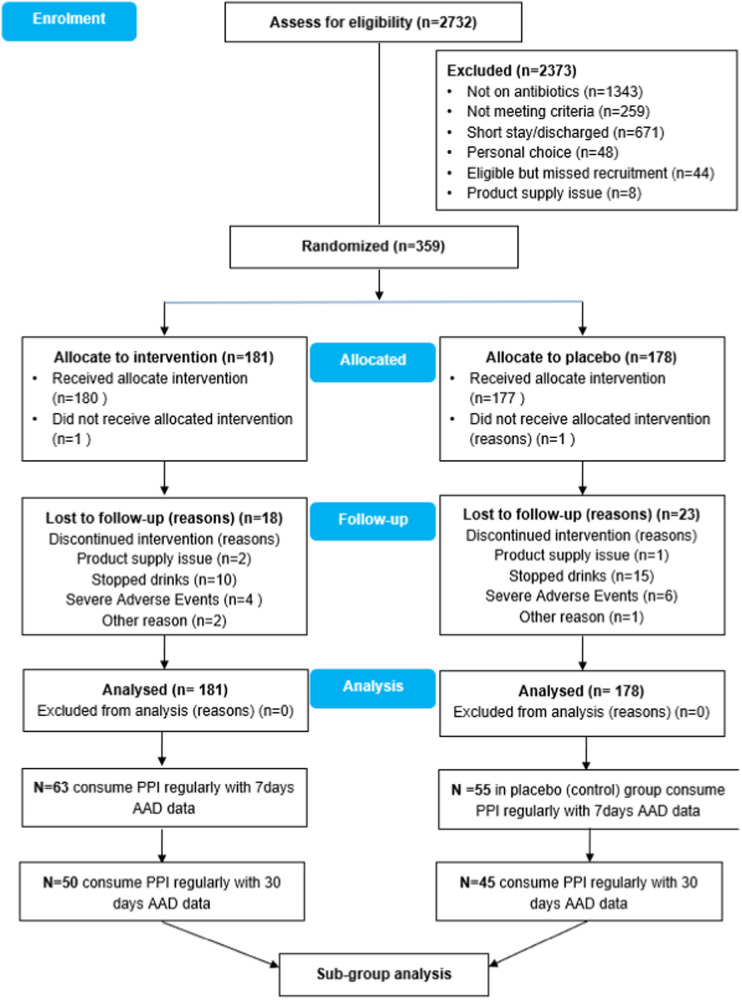
Trial Profile. Decision tree for recruitment and randomisation.

Over the 60 months of the study period, 359 patients were approached by the study team; 48 (11%) patients refused to participate in the study, 44 (9.6%) potentially eligible patients were missed because of the tight recruitment timeframe, and eight (1.7%) were excluded for logistical reasons. Of the 359 patients recruited into ECLISP study, 95 patients (27%) of patients met the inclusion of this sub-group analysis. The participants flow is summarised in Fig. 1.

The baseline characteristics of the 95 patients (median age: 57, IQ range: 25; 23% female; 88% Caucasian), and the outcomes are summarised in Table 1. SCI was traumatic in origin (*n* = 68, 67%) and non-traumatic in 27 case (28%). The median onset of SCI was 104 days (IQ range: 54–469).

**Table 1:** Baseline characteristics and outcomes summary.

	Total number with values (missing, %)	LcS group *n* = 50	Placebo group *n* = 45	*p*-value
Age Median (IQ range, range)	93 (2, 2.1%)	53 (28, 19–79)	59 (30, 19–86)	0.16
≥65 years (%)	93 (2, 2.1%)	13 (27%)	17 (39%)	0.30
Onset of SCI (days)	94 (1, 1.1%)	99 (437, 7– 20,013)	106 (411, 3–21,133)	0.90
Level of SCI: Tetraplegia (*n*, %)	95 (0, 0%)	24 (48%)	25 (56%)	0.46
Severity of initial neurological deficit				
AIS [15] grade A (*n*, %)	95 (0, 0%)	28 (56%)	15 (33%)	0.03
Study centre 1	95 (0, 0%)	26 (49%)	27 (51%)	
Study centre 2	95 (0, 0%)	21 (60%)	14 (40%)	
Study centre 3	95 (0, 0%)	3 (43%)	4 (57%)	
Mechanical ventilation (*n*, %)	95 (0, 0%)	5 (10%)	7 (16%)	0.42
Pressure ulcers (*n*, %)	95 (0, 0%)	15 (30%)	19 (42%)	0.21
History of previous ITU stay (*n*, %)	95 (0, 0%)	12 (24%)	13 (29%)	0.59
Number of drugs Median (IQ range, range)	95 (0, 0%)	8 (5, 4-20)	11 (3.5, 2–31)	0.69
Number of antibiotics (*n*, %)	95 (0. 0%)	1 (0.25, 1–7)	1 (1, 1–4)	0.16
Multiple antibiotics (*n*, %)	95 (0, 0%)	12 (24%)	19 (42%)	0.06
Duration of antibiotics Median (IQ range, range)	95 (0, 0%)	7 (5, 3–49)	8 (5, 3–38)	0.15
High risk antibiotics (*n*, %)	95 (0, 0%)	22 (44%)	29 (64%)	<0.05
Laxative use (*n*, %)	95 (0, 0%)	45 (90%)	39 (87%)	0.6
At undernutrition risk: SNST ≥ 11 (*n*, %)	95 (0, 0%)	22 (44%)	17 (38%)	0.54
Body mass index (BMI, kg/m^2^)	92 (3, 3.2%)	25.4 (8.0, 17–39)	25.3 (7.0, 17–47)	0.61
At overnutrition risk: BMI > 25 kg/m^2^ (*n*, %)	92 (3, 3.2%)	26 (53%)	25 (56%)	0.12
Obese: BMI > 30 kg/m^2^ (*n*, %)	92 (3, 3.2%)	10 (20%)	10 (22%)	0.79
Time to take first study drink after first antibiotic dose	95 (0, 0%)			
Within 24 h (*n*, %)	–	41 (82%)	37 (80%)	0.98
24–48 h (*n*, %)	–	9 (18)	9 (20%)	0.80

Data are *n* (%) unless otherwise stated. AIS = American Spinal Injury Association/International Spinal Cord Society neurological stand scale [11].

Number used to calculate proportions for other characteristics is proportion of patients with available follow up data.

*SCI* spinal cord injury, *SNST* Spinal Nutrition Screening Tool, *BMI* body mass index, *ITU* intensive therapy unit.

The prevalence of risk for undernutrition was 41% (*n* = 39) at the time of recruitment and 16 (17%) were malnourished on dietitian assessment.

Most participants (67%) received one antibiotic as their entry criterion to the study, but 21% received two, 7.4% received three and 4.2% received four or more antibiotics. A total of 19 different antibiotics were recorded in the present study: the oral route was used in 48% and the intravenous route was used in 52% of participants. The median length of antibiotic course was 7 days (IQ range: 6–11); no statistically significant differences was found with regard to nature or duration of antibiotic intake. The indications for antibiotic treatment were: urinary tract infections (*n* = 49, 47%), respiratory tract infections (*n* = 20, 19%), wound infections (8, 7.7%), pressure ulcer infections (7, 6.7%), post-operative infections (*n* = 4, 3.8%), sepsis (*n* = 4, 3.8%), eye infections (*n* = 2, 1.9%), surgical implant infection (*n* = 1, 0.9%) and others (*n* = 9, 8.6%).

At baseline, the LcS and placebo groups were similar with respect to demographic and clinical characteristics, which included: age, onset of SCI, those with tetraplegia, the percentage who were on mechanical ventilation, the percentage with pressure ulcers, the percentage with previous history of Intensive Therapy Unit (ITU) stay; laxative use; body mass index (BMI), the percentage of those nil-by-mouth and those with enteral feeding tubes (Table 1). The number of participants on high-risk antibiotics was higher in the placebo group (64% placebo group and 44% in LcS group, *p* < 0.05).

Two (2.1%) severe adverse events were reported in this sub-group analysis and they were not related to the use of investigational product (Table 2).

**Table 2:** Summary of severe adverse events.

	Total number with values (missing, %)	LcS group *n* = 50	Placebo group *n* = 45
Severe adverse events (%)	95 (0, 0%)	0% (0)	5.0% (2)
Unexpected SAE		0	2
Nature of SAE			
Transfer to other hospital (cardiology)		0	1
High dependency unit admission		0	1
Intensive care unit admission		0	0
Death		0	0

### Primary outcome

#### Antibiotic associated diarrhoea

**Fig. 2:**
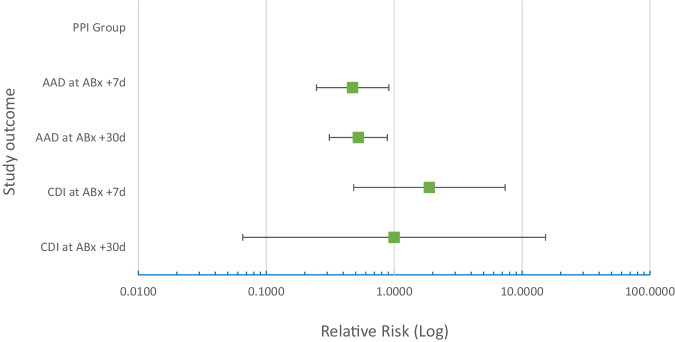
ECLISP outcome summary.

The overall prevalence of AAD was 40% at 30 days follow up. This is a statistically significant difference between the LcS and placebo groups (28 v 53%, RR: 0.53, 0.31–0.89; z = 2.5, *p* = 0.01) in regard to the intention to treat that included all patients with available end-point data (Fig. 2 and Table 3).

**Table 3:** ECLISP outcome measures summary.

Outcome measures	Total sample	LcS (Intervention) group			Placebo group			Absolute risk			Relative risk		
	(*n*, %)	(*n*, %)	95% LCI	95% UCI	(*n*, %)	95% LCI	95% UCI	ARR	(95% LCI)	(95% UCI)	RR	(95% LCI)	(95% UCI)
Primary outcome													
AAD – Abx + 30d	40% (38/95)	28% (14/50)	0.175	0.417	53.3% (24/45)	0.391	0.671	0.253	0.056	0.426	0.525	0.312	0.885
Secondary outcomes													
AAD – Abx + 7d	26.9% (32/119)	19.1% (12/63)	0.113	0.304	35.7% (20/56)	0.245	0.488	0.167	0.007	0.319	0.533	0.287	0.990
CDI – Abx + 7d	9.9% (8/81)	13.2% (5/38)	0.056	0.273	6.9% (3/43)	0.024	0.186	−0.062	−0.211	0.076	1.886	0.483	7.372
CDI – Abx + 30d	3.3% (2/6)	3.3% (1/30)	0.006	0.167	3.3% (1/30)	0.006	0.167	0.000	−0.136	0.136	1.000	0.066	15.260

AAD – Abx + 7d: occurrence of diarrhoea at 7 days after finished antibiotic course, 7 days after they stop intervention (LcS/placebo).

AAD – Abx + 30d: occurrence of diarrhoea at 30 days after finished antibiotic course, 23 days after they stop intervention (LcS/placebo).

CDI – Abx + 7d: occurrence of *C. diff* infection at 7 days after finished antibiotic course, 7 days after they stop intervention (LcS/placebo).

CDI – Abx + 30d: occurrence of *C. diff* infection at 30 days after finished antibiotic course, 23 days after they stop intervention (LcS/placebo).

### Secondary outcomes and predetermined subgroup analysis

There was a statistically significant difference between the LcS and placebo groups for the prevalence of AAD at 7 days follow up (19% v 36%, RR: 0.53, 95% CI: 0.29–0.99, z = 2.0; *p* = 0.04) but there was no significance observed in the duration of diarrhoea, the number of episodes of diarrhoea and the occurrence of CDI at 7 days follow up, nor at 30 days follow up for CDI (Table 3).

#### Risk factors for antibiotic associated diarrhoea/Clostridioides difficile infection

The risk factors for AAD at Abx + 30d were being in control group (taking placebo) and study site as the unique risk factors of AAD at 30 days follow up. The use of LcS was associated with a lower risk of AAD at 30 days follow up (28% v 53%, RR: 0.53, 95% CI: 0.31–0.89) (Table 4.1).

**Table 4:** 1: Risk factors for antibiotic associated diarrhoea/Clostridioides difficile infection: Primary outcome - occurrence of diarrhoea (AAD up to 30 days after antibiotic course is finished). 2: Risk factors for antibiotic associated diarrhoea/Clostridioides difficile infection: occurrence of diarrhoea (AAD up to 7 days after antibiotic course is finished).

1							
	Occurrence of diarrhoea upto 30 days after antibiotic course is finished (Log Reg) – Multivariate	B	S.E.	Sig.	Exp(B)	95% L. C.I. for EXP(B)	95% U. C.I. for EXP(B)
1	Age 65 or above	0.43	0.56	0.44	1.5	0.51	4.6
3	High risk antibiotics	0.91	0.56	0.10	2.5	0.83	7.3
4	Group Allocated: LcS	−1.0	0.52	<0.05	0.36	0.13	0.99
5	Centre (1, 2, 3) 1 vs 2	1.9	0.69	0.01	6.5	1.7	25
6	Centre (1, 2, 3) 1 vs 3	1.7	1.0	0.10	5.5	0.71	42
7	Route of antibiotics: IV	0.31	0.62	0.61	1.4	0.41	4.6
8	Number of antibiotics	0.11	0.34	0.74	1.1	0.58	2.2
9	Length of antibiotics included in trial (d)	0.01	0.04	0.89	1.0	0.93	1.1
0	Constant	−2.2	0.70	<0.01	0.11	0.03	0.42

	Occurrence of diarrhoea up to day 30 after antibiotic is finished *n* = 95		Total
	No	Yes	
Placebo control	21 (47%)	24 (53%)	45 (100%)
Intervention	36 (72%)	14 (28%)	50 (100%)
Total	57 (60%)	38 (40%)	95 (100%)

2							
	7 Day Dx (Log Reg) – Multivariate	B	S.E.	Sig.	Exp(B)	95% L. C.I. for EXP(B)	95% U. C.I. for EXP(B)
1	Age 65 or above	0.98	0.67	0.15	2.7	0.71	9.9
2	High risk antibiotics	1.8	0.72	0.01	6.2	1.5	25
3	Centre (1, 2, 3) 1 vs 2	2.1	1.1	0.05	7.8	0.99	62
4	Centre (1, 2, 3) 1 vs 3	4.1	1.4	<0.01	58	4.1	829
5	Level of SCI - tetraplegic vs paraplegic	1.6	0.95	0.09	4.9	0.76	31
6	High Tetraplegic (C1-C4, Y:1, N:0)	−0.30	0.85	0.72	0.74	0.14	3.9
7	Presence of pressure ulcers	1.3	0.81	0.12	3.5	0.72	17
8	Route of antibiotics: IV	−0.51	0.99	0.60	0.60	0.09	4.1
9	Additional line and tubes	1.8	1.04	0.09	5.8	0.75	44
10	Malnutrition Assessment: Yes	−0.41	0.85	0.63	0.66	0.13	3.5
11	At risk of undernutrition: SNST ≥ 11	−0.21	0.71	0.77	0.81	0.20	3.3
12	Number of drugs	0.24	0.10	0.01	1.3	1.1	1.5
13	Number of antibiotics	−0.09	0.34	0.80	0.92	0.47	1.8
14	Length of Antibiotics included in trial (day)	0.02	0.04	0.54	1.0	0.95	1.1
15	Additional fluid (ml)	0.00	0.00	0.33	1.0	1.0	1.0
0	Constant	−9.2	2.12	<0.01	0.00	0.00	0.00

	Occurrence of diarrhoea up to day 7 after antibiotics		Total
	No	Yes	
Placebo control	36 (642%)	20 (36%)	56 (100%)
Intervention	51 (81%)	12 (19%)	63 (100%)
Total	87 (73%)	32 (27%)	119 (100%)

B- Beta value for the predictor regression coefficient

SE of B is the standard error of the B coefficient.

Sig – is the Significance of the B coefficient.

Exp (B) is the Adjusted Odds Ratio produced by the B coefficient.

95% L. C.I. for EXP(B) is the 95% Lower confidence interval for the Adjusted Odds Ratio.

95% U. C.I. for EXP(B) is the 95% Upper confidence interval for the Adjusted Odds Ratio.

The multivariate logistic/regression analysis revealed independent risk factors for AAD at Abx + 7d: use of high-risk antibiotics (OR: 6.2; 95% CI: 1.5–25.4), study site (OR: 57.9; 95% CI: 4.1–829) and number of drugs (OR: 1.3; 95% CI: 1.1–1.5) (Table 4.2).

## Discussion

Optimisation of people with SCI’s experience in SCI rehabilitation and neurogenic bowel & bladder management has been a priority for both clinical [18] and research [19]. In 2014, the James Lind Alliance research priority setting exercise reported an improved bladder management, urinary tract infection and intervention in bowel management were listed within the top 10 research priorities for PWSCI [19].

People with SCI may be particularly vulnerable to diarrhoea and its consequences because of their long hospital stays for acute care and rehabilitation. The present study found the incidence of AAD similar to previous reports in the range of 15–36% [6–8]. but seems higher than studies conducted in general populations (11–18%) [20, 21]. This may be attributed to a longer follow-up period (30 days) than in many of the other published trials (often only 7–14 days) [20]. It is reported that diarrhoea may occur up to 2 months after discontinuing antibiotic treatment [22].

This sub-group analysis suggested that the LcS could reduce the risk of AAD in patients with SCI who consumed PPI regularly. These finding are similar to our earlier open-labelled study [6].

The present study defined AAD as ≥2 liquid stools (Bristol Stool Scale type 5, 6 or 7) for a 24-h period, whereas the previous trial required ≥3 days [6]. This altered definition may have led to a failure in distinction between clinically relevant AAD and loose stool due to neurogenic bowel as a result of SCI. Indeed, the definition of AAD varies widely between published studies. For example, Rajkumar et al. [20] defined diarrhoea as ≥2 loose stools, Bristol 6 or 7 a day for ≥3 days, whereas Allen et al. [21] and Helps et al. [23] defined diarrhoea from ≥3 loose stools, Bristol 5, 6 or 7 in a single 24-h period. The definition for CDI also varies. The use of standardised definitions of AAD/CDI will greatly improve the quality and the interpretation of newer research studies, especially important for systematic reviews and meta-analyses.

There are some limitations in this study. The inclusion of patients treated in different SCI centres could be considered a strength, but can also be regarded as a weakness. Infection control policies, AAD/CDI definition were different in the participating SCI centres, thus the influence of these factors on the study results could not be excluded. In addition, different SCI centre may have different policies on antibiotic prescribing and different catheters and bowel management programme. Nevertheless, the selection of the SCI centres was at the discretion of the authors; and those selected represented approximately 45–50% of the SCI centre beds in the UK, therefore, we would assume the result derived from this study can be considered representative.

The present study did not judge whether antibiotics were prescribed appropriately; there may be differences between centres in their antibiotics prescribing and bowel management programme.

Current evidence remains equivocal in whether probiotics could reduce the incidence of AAD/CDI either in the general hospitalised or SCI populations [20, 21, 24]. The complexity of probiotic use is not just strain-, product-, dose- and disease specific, but also includes defining when the probiotic should be administered and the duration of its use; all these factors need to be considered. The present study dose of a minimum of 6.5 ×10^9^ CFU LcS was selected based on the previous trial’s data [6]. LcS is well tolerated in clinical settings and has been used in a broad range of patients [25, 26]. However, dose and type of probiotic vary between published studies. For example, Allen et al. [21] used a mixed strains probiotic (*L. acidophilus* CUL60, CUL21, *B. bifidum* CUL20, *B. lactis* CUL34 in 6 ×10^9^ CFU/day), Helps et al. [23] used a single strain probiotic (LcS in 13 ×10^9^ CFU/day), and Rajkumar et al. [20] used mixed strains (*L. casei* immunitas DN-114001 in 10 ×10^9^ CFU/day) as did Selinger et al. [27] (VSL#3 in 900 ×10^9^ CFU/day). It has been suggested that the dose of probiotic should >10^10^ CFU/ day in order to prevent AAD [28]. The dose of 6.5 ×10^9^ CFU LcS was selected on pragmatically to limit the volume to one bottle to aid compliance, and it is possible that a higher does could have yielded even greater benefits. However, the efficacy of increasing the dose of the probiotic LcS should be carefully monitored to avoid unexpected adverse effect.

The compliance with LcS therapy in the present study was good (92%), with no known adverse events directly related to LcS.

Another important criterion for any probiotic used is that the strains survive the passage through the stomach and arrive in a viable state in the small intestine and colon. LcS has been shown to survive and is well tolerated in the upper gastrointestinal tract and reach the large intestine in a viable state [29, 30].

Due to global concern of antibiotic resistance as infections become drug resistant and antibiotics becoming less effective at treating infections, and in an effort to prevent such occurrence, we assume that a non-antibiotic therapy, such as probiotic supplement is highly desirable if this could prevent AAD/CDI. Indeed, reducing antibiotic use could improve quality of life, save money and help preserve the usefulness of existing antibiotics.

For gastric protection in the acute phase after SCI, approximately 1 in 3 people are prescribed PPI. We believe that the number of patients prescribed PPI instead is much higher due to national shortage of H_2_ blocker. The present sub-group analysis suggests that the daily consumption of probiotic LcS has the potential to prevent AAD in the higher risk group patients on regular PPI. In order to translate into improved clinical outcome, a confirmatory randomised, placebo-controlled study is recommended to confirm this apparent therapeutic success.

### Supplementary information

Supplementary File 1

Supplementary File 2

Supplementary File 3

## Data Availability

The study is registered with ISRCTN, number: 13119162. In accordance with the protocol, no investigators will have access to participant data with identifiers. De-identified participant data that underlie the results reported in this article will be made available as supplementary material.
