# Supplementary material for: Lactobacillus casei Shirota probiotic drinks reduce antibiotic associated diarrhoea in patients with spinal cord injuries who regularly consume proton pump inhibitors: a subgroup analysis of the ECLISP multicentre RCT
Source: Spinal Cord. 2024 Mar 22;62(5):255–63. doi: 10.1038/s41393-024-00983-w (PMC11176055; doi:10.1038/s41393-024-00983-w)
Supplement: Supplementary file 1 [file 41393_2024_983_MOESM1_ESM.pdf]

Efficacy of **C**onsuming **L**cS **I**n **S**pinal cord injury **P**atients (**ECLISP**)

Effect of *Lactobacillus casei* Shirota in preventing antibiotic associated diarrhoea (AAD) including *Clostridium difficile* associated diarrhoea (CDAD) in patients with spinal cord injuries: a multicentre, randomised, double-blind, placebo-controlled trial

---

Clinical Research Protocol
[10.03.2017] *CRP_v 3.2*

REC reference number: 14/SC/1101
ISRCTN reference number: 13119162

Principal Investigator: Dr. Samford Wong[1,2,3]

Co- Investigators: Dr. Ali Jamous[1], Dr. Jean O'Driscoll[4], Dr. Ravi Sekhar[5], Dr. Shashi P. Hirani[3], Dr. Graham Harvey[6], Mr. Naveen Kumar[6], Dr. Ramaswamy Hariharan[7], Dr Anand Viswanathan, Prof. Alastair Forbes[2]

[1]National Spinal Injury Centre, Stoke Mandeville Hospital, Aylesbury, HP21 8AL
[2] Centre for Gastroenterology & Clinical Nutrition, University College Hospital
[3] Health Service Research, City University London, London EC1V 0HB
[4] Dept. of Microbiology, Stoke Mandeville Hospital, Aylesbury, HP21 8AL
[5] Dept. of Gastroenterology, Stoke Mandeville Hospital, Aylesbury HP21 8AL
[6] Midland Centre for Spinal Injury, Robert Jones and Agnes Hunt Orthopaedic Hospital NHS foundation Trust, Oswestry SY10 7AG
[7] The Princess Royal Spinal Injuries Centre, Northern General Hospital, Herries Road, Sheffield, S5 7AU

Version Number: ECLISP_v 3.2 Date: 08-05-2018
Financial Sponsor study number: 13UK-1-SMH-AAD2

Confidentiality statement:
The information provided in this document is strictly confidential and is available for review to investigators, potential investigators and appropriate Ethics Committees or Investigational Review Boards. No disclosure should take place without written authorisation from the Sponsor, the Financial Sponsor and Principal Coordinating Investigator Dr S. Wong, except to the extent necessary to obtain informed consent from potential participants.

Table of contents

1 Protocol synopsis 4
2 General information / responsibilities 7
2.1 Responsibilities 7
2.2 Study Sites 11
2.3 Trial Registration 12
3 Rationale of the Study & Definitions 12
3.1 Definitions 13
4 Primary and secondary objectives 13
4.1 Primary Objective 13
4.2 Secondary Objectives 13
5 Trial design 14
5.1 Study population 14
5.1.1 General Inclusion Criteria 14
5.1.2 General Exclusion Criteria 14
5.2 Recruitment of participants / Screening 14
5.3 Informed consent procedure 15
5.4 Investigational product and comparator 15
5.4.1 Investigational product 15
5.4.2 Comparator 16
5.5 Study phases & Timeline 16
5.5.1 Recruitment & Informed Consent 16
5.5.2 Study procedure: opening assessments 16
5.5.3 Randomisation: blinded treatment. Group allocation 17
5.5.4 Product Delivery 17
5.5.5 Administration of intervention 18
5.5.6 End of Intervention; Start of Follow-up 19
5.5.7 Study endpoint / closing assessment 19
5.5.8 Logistics Stool Specimen 20
6. Definitions of outcomes and study variables 22
6.1 Primary outcome: Occurrence of antibiotic-associated diarrhoea (AAD) 22
6.2 Secondary outcomes 22
6.2.1 Incidence of C. difficile diarrhoea (CDAD) 22
6.2.2 Duration of diarrhoea 22

6.2.4 Change in gut microbiota (stool specimen analysis) *22*
6.2.5 Quality of life *22*
6.3 Outcome assessors 22
7 Statistical planning 22
7.1 Hypothesis 22
7.2 Sample size considerations 23
7.3 Sample per Investigative Site 23
7.4 Statistical analysis 23
7.5 Interim Analysis 24
8. Patient risk analysis 24
8.1 Risks 24
8.2 Benefits 25
9 Adverse Event reporting 25
9.1 Adverse Events 25
9.2 Serious Adverse Events (SAE) 25
9.3 Product-related anticipated adverse events 26
9.4 Adverse event reporting 26
10 Data management 26
10.1 Data collection, source data, storage and archiving 26
10.2 Confidentiality 27
11 Study Management and Quality Control 28
12 Regulatory aspects 28
13 Study report and publication policy 29
13.1 Final report 29
13.2 Publication & Presentation 29
14 Termination criteria 29
14.1 Premature Termination of the Procedure 29
14.2 Removal of Patients from the study 29
14.3 Termination of the study 29
15 Time schedule 30
16 Finances 31
17 Appendices 32
18 References 37

3 Protocol synopsis

**Official title**	Effect of *Lactobacillus casei* Shirota in preventing antibiotic associated diarrhoea (AAD) including *Clostridium difficile* associated diarrhoea (CDAD) in patients with spinal cord injuries: a multicentre, randomised, double-blind, placebo-controlled trial
**Short title**	**E**fficacy of **C**onsuming **L**cS **i**n preventing AAD / CDAD **I**n **S**pinal cord injury **P**atients (**ECLISP**)
**Protocol version**	**3.2 (08 May 2018)**
**Sponsor**	Buckinghamshire Healthcare NHS Trust (BHT)
**Financial Sponsor**	Yakult Honsha Co Ltd (YHL)
**Product and placebo**	Supplied by Yakult Europe B.V. (YEU)
**CRO**	None
**Trial registration**	ISRCTN: 13119162
**Project purpose**	To assess the efficacy of a probiotic containing a minimum of 6.5x 10^9 *Lactobacillus casei* Shirota for the prevention antibiotic-associated diarrhoea (AAD) including *Clostridium difficile* associated diarrhoea (CDAD).
**Condition**	Spinal Cord Injury (SCI) patients who are newly started on antibiotics
**Intervention**	One bottle of Yakult containing 6.5 x 10^9 *Lactobacillus casei* Shirota once a day during the course of antibiotics and for 7 days after the course finishes.
**Comparison**	An identical looking placebo once a day during the course of antibiotics and for 7 days after the course finishes.
**Evaluation type**	Experimental study
**Study design**	A multicentre, randomised, double-blind, placebo-controlled trial
**Study site(s)**	National Spinal Injuries Centre (NSIC), Aylesbury, United Kingdom HP21 8AL<br><br>The Midlands Centre for Spinal Injury, The Robert Jones & Agnes Hunt Orthopaedic Hospital, Oswestry, SY10 7AG<br><br>The Princess Royal Spinal Injuries Centre, Northern General Hospital, Herries Road, Sheffield, S5 7AU

**Principal Coordinating Investigator (PCI)**	Dr. Samford Wong Clinical Lead Dietitian – Research / Spinal Injuries, NSIC
**Primary objective**	To assess the efficacy of a probiotic preparation (Yakult) containing a minimum of 6.5 x 10^9 *Lactobacillus casei* Shirota (LcS) compared to placebo for the prevention of AAD
**Secondary objective**	Analyse the effect of LcS on (i) occurrence of C. difficile diarrhoea; (ii) duration of diarrhoea; (iii) gastrointestinal microbiota and (iv) quality of life
**Hypothesis**	It is hypothesised that maintenance of a healthy commensal microbiota during antibiotic treatment by administration of a daily probiotic (*Lactobacillus casei* Shirota, as Yakult) will significantly reduce the occurrence of AAD and CDAD, and thus improve quality of life in spinal cord injury patients over time in comparison to the placebo control
**Primary endpoint**	Occurrence of antibiotic-associated diarrhoea (i.e. 2 or more loose stools, Bristol Stool Scale type 5 or above) up to 30 days after finishing probiotic/placebo
**Secondary endpoints**	• Incidence of *C. difficile* diarrhoea [CDAD], as diagnosed by a positive *C. difficile* toxin test and stool culture on all patients with undiagnosed diarrhoea.<br>• Duration of diarrhoea (i.e. 2 or more loose stools, Bristol Stool Scale type 5 or above).<br>• Change in prevalence of *C. difficile* toxin genes.<br>• Change in gut microbiota (stool specimen analysis by TaqMan PCR)<br>• Quality of life by WHO-BREF.<br>• To determine if malnutrition (undernutrition) is a risk factor for AAD and CDAD<br>• To determine if use of proton pump inhibitors (PPI) is a risk factor for AAD and CDAD

**Statistical considerations and estimated enrolment**	Power calculation: Based on pilot data, this study aimed to find a difference of 17.9% in the proportion of patients with diarrhoea in two groups, (i) those on proton pump inhibitors – 56.3% and (ii) those on LcS – 38.4%. With α=0.05 and power of 90%, we estimate a sample size of 162 per group. After accounting for dropouts at 10% (across the length of the trial) this will require 180 per arm (total n=360).<br>Fisher's exact test and χ^2 test will be used to compare rates of diarrhoea, as well as rates of AAD and CDAD. Relative risk and the number needed to treat, both with 95% confidence intervals, will be used to describe the treatment effect of probiotic.
**Expected Study start date**	Q4 2014
**Estimated study completion**	Q3 2018
**Eligibility inclusion criteria**	• Adult (≥18 years)<br>• Patient is likely to remain in the SCI centre for more than 6 weeks can be included.<br>• Newly started oral or intravenous antibiotics (the course of antibiotics used as treatment for an infection should be a minimum of 3 days) (single or multiple)<br>• Able to take study drinks within 48 hours of first dose of antibiotics.

**Eligibility exclusion criteria**	• Re-recruit patient (ie, only patients recruited for the first time are allowed)<br>• Antibiotic use in the 30 days prior to the study product first administration apart from a single dose of prophylactic given more than 14 days before the study product's first administration.<br>• Diarrhoea within the proceeding 7 days prior to commencement of intervention<br>• Bowel pathology that could result in diarrhoea<br>• Recent bowel surgery<br>• Infective endocarditis<br>• Active inflammatory bowel disease<br>• Pancreatitis<br>• Regular probiotic use<br>• Any illness requiring ITU intervention or systemic sepsis<br>• Immunosuppression<br>• Nil-by-mouth status for any reason<br>• Non–functioning gut<br>• Known cows' milk protein intolerance<br>• Psychiatric or cognitive conditions that may interfere with the study<br>• Patients incapable of providing informed consent due to a mental incapacity<br>• Patients unlikely to comply with study requirements<br>• Pregnant or breastfeeding women<br>• Prisoners
**NHS strategic health authority**	Buckinghamshire Healthcare NHS Trust, NHS South Central
**Insurance / Indemnity Scheme**	NHS indemnity scheme will apply

2 General information / responsibilities

2.1 Responsibilities

Principal Investigator

Dr Samford Wong<br>*Clinical Lead Dietitian: Spinal Injuries/Research*<br>Role: protocol development; collection; management; analysis / interpretation of data; writing of report	National Spinal Injuries Centre<br>Buckinghamshire Healthcare NHS Trust<br>HP21 8AL, Aylesbury, Buckinghamshire, UK<br>☏ +44 1296 31 5529<br>E-Mail: Samford.Wong1@nhs.net

Co- Investigators

Dr Ali Jamous<br>Consultant Surgeon in Spinal Cord Injury<br><br>Role: protocol development; clinical supervision; writing of report	International Spinal Injuries Rehabilitation Centre<br>Royal Buckinghamshire Hospital<br>HP19 9AB, Aylesbury, Buckinghamshire, UK<br>☏ +44 1296 678800<br>E-Mail: Ali.Jamous@btinternet.com
Dr. Jean O'Driscoll<br>Consultant Microbiologist and Director of Infection Prevention and Control<br><br>Role: protocol development, validation and guardian of laboratory data, manuscript revision	Department of Microbiology<br>Stoke Mandeville Hospital<br>Buckinghamshire Healthcare NHS Trust<br>Aylesbury<br>HP21 8AL<br>E-Mail: jean.odriscoll1@nhs.net
Dr. Ravi Sekhar<br>Consultant Gastroenterologist<br><br>Role: protocol development, manuscript revision	Department of Gastroenterology<br>Stoke Mandeville Hospital<br>Buckinghamshire Healthcare NHS Trust<br>Aylesbury<br>HP21 8AL<br>E-Mail: rsekhar@nhs.net
Dr. Shashivadan P Hirani<br>Data Analyst / Statistical Adviser<br>Senior Lecturer in Health Services Research & Health Psychology<br><br>Role: protocol development, data analysis / statistical adviser; manuscript revision	Health Service Research,<br>City University London<br>College Building, Northampton Square<br>London EC1V 0HB<br>☏ +44 (0) 20 7 040 0880<br>E-Mail: Shashi.hirani.1@city.ac.uk
Mr Nigel Henderson<br>Clinical Director NSIC<br><br>Role: ensure the proper delivery of the study	National Spinal Injuries Centre<br>Stoke Mandeville Hospital<br>Mandeville Road<br>Aylesbury<br>HP21 8AL<br>E-mail: nigel.henderson@buckshealthcare.nhs.uk
Dr. Graham Harvey<br>Consultant Microbiologist<br><br>Role: protocol development and guardian of laboratory data (Oswestry), manuscript revision	The Midlands Centre for Spinal Injury<br>The Robert Jones & Agnes Hunt Orthopaedic Hospital<br>SY10 7AG, Oswestry, UK<br>☏ +44 1691 404650<br>E-Mail: Graham.Harvey@sath.nhs.uk

Mr. Naveen Kumar<br>Consultant Surgeon in Spinal Injuries & Rehabilitation Medicine<br><br>Role: protocol development; clinical supervision; revision of report	Midland Centre for Spinal Injuries<br>The Robert Jones and Agnes Hunt Orthopaedic Hospital NHS Foundation Trust<br>Oswestry, SY10 7AG<br>☏ +44 1691 404646<br>Email: naveen.kumar@rjah.nhs.uk
Dr. Ramaswamy Hariharan<br>Consultant in Spinal Injuries<br><br>Role: protocol development; clinical supervision; revision of report	The Princess Royal Spinal Injuries Unit<br>Northern General Hospital<br>Herries Road<br>Sheffield, S5 7AU<br>☏ +44 (0114) 2715658<br>Email: ram.hariharan@sth.nhs.uk
Dr. Anand Viswanathan<br>Clinical Fellow<br><br>Role: protocol development, data collection, revision of report	The Princess Royal Spinal Injuries Unit<br>Northern General Hospital<br>Herries Road<br>Sheffiled, S5 7AU<br>☏ +44 (0114) 2715658<br>Email: anand.viswanathan @sth.nhs.uk
Prof. Alastair Forbes<br>Clinical Professor in Medicine and Chief of Research and Innovation<br><br>Role: protocol development; data interpretation, manuscript revision and guarantor of the paper	Norwich Medical School<br>University of East Anglia<br>Bob Champion Research & Education Bldg 2.12<br>NR4 7TJ<br>☏ 01603 591903<br>E-Mail: alastair.forbes@uea.ac.uk

Study Coordinator:

Research Fellow<br>Alka Pandey<br>Research Fellow / Specialist Dietitian<br><br>Interim contact<br>Janine Turner	National Spinal Injuries Centre<br>Research Office A22<br>Stoke Mandeville Hospital<br>HP21 8AL, Aylesbury, Buckinghamshire, UK<br>☏ +44 1296 31 5775<br>E-Mail: Alka.pandey@nhs.net<br><br>☏ +44 1296 31 5529<br>E-Mail: Janine.turner3@nhs.net

Theresa Garratt<br>Research Nurse	The Midlands Centre for Spinal Injury<br>The Robert Jones & Agnes Hunt Orthopaedic Hospital<br>SY10 7AG, Oswestry, UK<br>☏ +44 1691 404139; +44 07746502994<br>E-Mail: Theresa.Garratt@rjah.nhs.uk
Lissie Webster<br>Research Nurse	The Princess Royal Spinal Injuries Unit<br>Northern General Hospital<br>Herries Road<br>Sheffield, S5 7AU<br>☏ +44 (0114) 2715618<br>E-mail: lissie.webster@sth.nhs.uk

R&D Manager

Denise Watson<br>Research & Innovation Manager	Department of Research & Development<br>Buckinghamshire Healthcare NHS Trust<br>HP21 8AL, Aylesbury, Buckinghamshire, UK<br>☏ +44 1296 31 6065<br>E-Mail: denise.watson@buckshealthcare.nhs.uk
Teresa Jones<br>Research Manager	Department of Research & Development<br>ARC Building<br>The Robert Jones & Agnes Hunt<br>Orthopaedic Hospital NHS Foundation Trust<br>Oswestry<br>SY10 7AG<br>E-mail: teresa.jones@rjah.nhs.uk
Dr Debby Hawkins<br>Research Co-ordinator<br><br>Aimee Card<br>R&D Co-ordinator<br>(role: provide final C&C approval)	The Princess Royal Spinal Injuries Centre,<br>Northern General Hospital, Herries Road,<br>Sheffield, S5 7AU<br>☏ +44 (0114) 3052249<br>Fax: 0114 2715649<br>E-mail: Debby.Hawkins@sth.nhs.uk<br><br>Clinical Research Office, D Floor, Royal Hallamshire Hospital, Glossop Road, Sheffield.<br>☏+44 (0114) 2265945<br>E-mail: aimee.card@sth.nhs.uk

Additional microbiological analysis

Yakult Honsha European Research Center for Microbiology ESV<br>[YHER]	Technologiepark 4, 9052 Gent-Zwijnaarde, Belgium<br>BTW: BE 0873.953.667<br><br>Contact person: Dr Kazunori Matsuda<br>Tel: +32 (0)9 241 11 34<br>Fax: +32 (0)9 241 11 33<br>E-mail: kazunori.matsuda@yher.be

Financial Sponsor (and contact person)

Yakult Honsha Co. Ltd<br>[YHL]	1-19, 1-chome, Higashi-Shimbashi<br>Minato-ku 105-8660, Tokyo, JAPAN<br><br>Contact person: Dr Toshihisa Ota

Financial Sponsor's contact persons in Europe

Yakult Europe BV<br>[YEU]	Dr. Kaori Suzuki (YEU)<br>Manager Human Study<br>Yakult Europe B.V.<br>Schutsluisweg 1<br>1332 EN Almere<br>The Netherlands<br>E-Mail: KSuzuki@yakulteurope.com
Yakult UK Ltd.<br>[YUK]	Dr Louise Wilson (YUK)<br>Assistant Science Manager<br>Yakult UK Ltd,<br>Artemis, Odyssey Business Park, West End Rd<br>HA4 6QF, South Ruislip, Middlesex, UK<br>☎ +44 20 8842617<br>🖷: +44 20 88393250<br>E-Mail: LWilson@yakult.co.uk

Independent consultant / external monitor to the study

PharmExcel Ltd	Yvanne Enever, MROPRA, MICR, MRQA<br>Venture House<br>6 Silver Court<br>Watchmead<br>Welwyn Garden City<br>Hertfordshire<br>AL7 1TS<br>Main Line: +44 (0) 1707 290477<br>Mobile: +44 (0)7810750626<br>E-Mail: yvanne.enever@pharmexcel.co.uk

2.2 Study Sites

This study will be conducted at the following 3 British SCI centres:

Principal Centre:
National Spinal Injuries Centre (one of the eleven spinal injuries centres in the UK)
Stoke Mandeville Hospital
Buckinghamshire Healthcare NHS Trust
HP21 8AL, Aylesbury, Buckinghamshire, UK

Centre size: 115 beds
Estimate recruitment: 8 patients per month, 96 patients per year

Centre 2:
The Midlands Centre for Spinal Injury (one of the eleven spinal injuries centres in the UK)
The Robert Jones & Agnes Hunt Orthopaedic Hospital
SY10 7AG, Oswestry, UK

Centre size: 44 beds
Estimate recruitment: 3 patients per month, 36 patients per year

Centre 3:
The Princess Royal Spinal Injuries Centre (one of the eleven spinal injuries centres in the UK)
Northern General Hospital
Herries Road, Sheffield, S5 7AU

Centre size: 60 beds (approx. 20 beds for acute admissions)
Estimate recruitment: 3 patients per month, 36 patients per year

Total UK SCI beds: 476 beds http://www.bascis.org.uk

2.3 Trial Registration

ClinicalTrials.gov: Identifier: ISRCTN: 13119162

3 Rationale of the Study & Definitions

Probiotics, defined as '*live microorganisms which, when administered in adequate amounts, confer a health benefit on the host*' (FAO/WHO, 2002), have been proposed to help maintain a healthy gut microbiota in hospitalised patients on antibiotic therapy, particularly those on broad spectrum antibiotics. It is hypothesised that maintenance of a

healthy commensal microbiota during antibiotic treatment by administration of a daily probiotic (*Lactobacillus casei* Shirota, as Yakult) will significantly reduce the occurrence of AAD and CDAD, and thus improve quality of life in spinal cord injury patients over time in comparison to the placebo control. A previous trial in patients with spinal cord injuries (SCI) suggests that a probiotic preparation (*Lactobacillus casei* Shirota: LcS) can prevent antibiotic associated diarrhoea (AAD) (54.9% v 17.1%) (Wong et al, 2014).

Apart from the above open-labelled study conducted at NSIC at Stoke Mandeville Hospital, other studies have reported Yakult effectiveness in reducing antibiotic-associated diarrhoea (Martinez *et al* 2003; Pirker *et al* 2013; Lewis *et al* 2009). The open-labelled study conducted in the NSIC from 2010 to 2012 showed that one bottle of Yakult during antibiotics and for one week after the medication ceased, was significantly effective in reducing incidence of AAD. However, in order to confirm these effects and fully demonstrate the extent of LcS effectiveness in these patients, a larger placebo-controlled study including different geographical locations is indicated.

3.1 Definitions

Baseline is defined as all measurements taken within the week before administration of the first intervention.
Intervention is defined as the administration of verum (Yakult) or placebo.
Verum is defined as one bottle of Yakult containing a minimum of 6.5 x 10^9 *Lactobacillus casei* Shirota once a day during a course of antibiotics and for 7 days after the course finishes.
Placebo is defined as a drink with identical taste and appearance to the test intervention but not containing any bacteria (including *Lactobacillus casei* Shirota), once a day during a course of antibiotics and for 7 days after the course finishes.
Study start point is defined as the first day of administration of the first intervention.
Study endpoint is defined as the last day following 30 days of follow-up after finishing administration of the intervention.

4 Primary and secondary objectives

4.1 Primary Objective

To assess the efficacy of a probiotic preparation (Yakult) containing a minimum of 6.5 x 10^9 *Lactobacillus casei* Shirota (LcS) compared to placebo for the prevention of antibiotic-associated diarrhoea (AAD).

4.2 Secondary Objectives

To analyse the effect of LcS on the
- Occurrence of *C. difficile* diarrhoea (CDAD)
- Duration of diarrhoea
- Change in gastrointestinal microbiota (stool specimen analysis)
- Quality of life

- To determine if malnutrition (undernutrition) is a risk factor for AAD and CDAD
- To determine if use of proton pump inhibitors (PPI) is a risk factor for AAD and CDAD.

5 Trial design

This is a multicentre, randomised, double-blind, placebo-controlled, parallel trial to be conducted in three specialised Spinal Cord Injury Centres in the United Kingdom. See Appendix 5 for the decision tree outlining recruitment and randomisation of subjects.

5.1 Study population

5.1.1 General Inclusion Criteria

- Adult (≥18 years)
- Patient is likely to remain in the SCI centre for more than 6 weeks can be included
- Newly started antibiotics (the course of antibiotics used as treatment for an infection should be a minimum of 3 days) (single or multiple)
- Able to take study drinks within 48 hours of first dose of antibiotics

5.1.2 General Exclusion Criteria

- Re-recruit patient (ie, only patients recruited for the first time are allowed)
- Antibiotic use in the 30 days prior to the study product first administration apart from a <u>single</u> dose of prophylactic given more than 14 days before the study product's first administration.
- Diarrhoea within the preceding 7 days prior to commencement of intervention
- Bowel pathology that could result in diarrhoea
- Recent bowel surgery
- Infective endocarditis
- Active inflammatory bowel disease
- Pancreatitis
- Regular probiotic use
- Any illness requiring ITU intervention or systemic sepsis
- Immunosuppression
- Nil-by-mouth status for any reason
- Non–functioning gut (Include those on Parenteral Nutrition)
- Known cows' milk protein intolerance
- Psychiatric or cognitive conditions that may interfere with the study
- Patients incapable of providing informed consent due to a mental incapacity
- Patients unlikely to comply with study requirements
- Pregnant or breastfeeding women
- Prisoners

5.2 Recruitment of participants / Screening

An invitation letter from potential participant's consultant and a participant information sheet will be sent to all patients with SCI admitted to the SCI centre upon admission. A member of study team will be available to answer any study queries upon request.

All admitted patients reviewed for potential consideration for the study and which are excluded, will be logged on the pre-screening log maintained by the study team.

Further details on the general process for obtaining informed consent can be found under *Item 5.3*.

5.3 Informed consent procedure

The study team will be contacted by the ward and informed when a patient is to start / or has been started on antibiotic therapy for an infection. A member of the research team will approach the subject and provide the participant information sheet and if the subject shows an initial interest, the study coordinator will explain (1) the purpose of the study, (2) the procedures, (3) the risk / benefits, (4) alternatives to participation, (5) and the confidentiality. At this stage, the study coordinator will emphasise that the current feasibility study does not influence the provision of other healthcare services delivered by the specialised services. Candidate will be offered up to 24 hours to consider participation in the current study and will have the opportunity to ask any study-related questions that arise. Should the patient wish to go ahead with the study, consent will be specifically sought for 1. participation in this study and 2. the use and storage of de-identified data for the current research project.

Patients able to consent for themselves will be asked to sign an informed consent form. A witness will be asked to sign on behalf of those patients unable to sign the form due to physical disabilities. After obtaining informed consent, patients will be allocated (SM xx, Os xx, or Ts xx depending on sites), undergo their baseline assessments and offered Yakult or placebo (according to randomisation result) within 48 hours of commencing their antibiotic. The study drink will then be given once a day at the drug round in accordance with the protocol.

A master ID log will be maintained at each site.

The participant will be informed about their right to withdraw from the study at any time and for any reason without sanction, penalty, or loss of benefits to which the patient is otherwise entitled. A register will be kept of all patients who withdraw from the study. The reason for consent withdrawal will be documented along with the following core patient characteristics: age, gender and level and severity of spinal cord injury.

A copy of the signed Informed Consent Form - approved by the Research Ethics Committee - will be retained at the investigational site along with other investigational forms. A copy of the signed informed consent form will be placed into the patient's medical record, patient file (original) and a copy will be handed over to the patient.

5.4 Investigational product and comparator

5.4.1 Investigational product

Yakult containing a minimum of 6.5x 10^9 live cells of *Lactobacillus casei* Shirota.

Subjects will receive one bottle of Yakult (a fermented milk drink containing the probiotic bacterium *Lactobacillus casei* Shirota) or an identical looking placebo, once daily during a

course of antibiotics and for 7 days after the course finishes after which participants will be followed for four weeks. The study intervention must be given within 48 hours of the patient commencing antibiotics.
Yakult and placebo will be supplied as fermented milk in sealed pots of 65 ml with date stamped expiry. They should be stored at approximately 7 degrees Celsius (domestic refrigerator). By the time of delivery, the products will have a shelf life of just over two weeks, so fresh supplies will be delivered every two weeks.

Ingredients (Yakult): Water, skimmed milk (reconstituted), glucose-fructose syrup, sugar, maltodextrin, flavouring, contains *Lactobacillus casei* Shirota.

Nutritional Values	Per 100ml	Per bottle (65 ml)
Energy	280.0 kJ / 66.0 kcal	182.0 kJ / 42.9 kcal
Protein	1.3 g	0.9 g
Carbohydrate	14.7 g	9.6 g
Of which sugars	14.2 g	9.2 g
Fat	< 0.1 g	< 0.1 g
Of which saturates	< 0.1 g	< 0.1 g
Fibre	0 g	0 g
Sodium	0.02 g	0.01 g

5.4.2 Comparator

The placebo drink has similar packaging, taste and nutritional content, but does not contain any *L. casei* Shirota.

5.5 Study phases & Timeline

A schematic representation of the study phases & timeline is presented in *Appendix 1: Study flow chart*.

5.5.1 Recruitment & Informed Consent

See *headings 5.2 and 5.3* for details.

5.5.2 Study procedure: opening assessments

After having obtained informed consent (1x copy to patients; 1x copy in medical notes; 1 x original in the investigator site file), basic patient's clinical characteristics and baseline measurements, as outlined on the Case Report Form (CRF, see *Item 12.1*), will be collected. No intervention will be administered prior to the completion of the study opening visit.

- Baseline demographics and clinical data
- Record use of medications (including use of antibiotics, laxatives, anti-diarrhoeal agents, proton pump inhibitors, H_2 blocker)
- Nutrition risk scores – Spinal Nutrition Screening Tool (Wong et al, 2010, 2012)

- Quality of Life evaluation – WHO-QoL BREF (http://www.who.int/substance_abuse/research_tools/en/english_whoqol.pdf)
- Bowels function measures – stool type, duration of diarrhoea
  Definition of diarrhoea: two or more loose stools, Bristol Stool Scale (O'Donnell LD,1990) type 5 or above.
  Constipation: using Rome III criteria: Functional constipation http://www.romecriteria.org/assets/pdf/19_RomeIII_apA_885-898.pdf (Drossman DA, 2006).
- Stool Specimen analysis (Kubota *et al.* 2014)
  One sample will be taken, frozen and stored frozen and then sent to YHER for TaqMan PCR analysis of toxigenic *C. difficile.* This analysis will be dependent on available resources at YHER. Further microbial analysis may also be done. This will be in the participant information sheet and on the consent form.

5.5.3 Randomisation: blinded treatment. Group allocation

Following enrolment, participants will be randomised to either the intervention or placebo group. Random assignment will be performed by Dr. Shashi Hirani. To ensure balanced groups for interim safety analysis, we will utilise a block randomisation technique, with permuted blocks of 30 for each study centre and for previous antibiotic use (15 to group 1 and 15 to group 2 per block), until sample size criteria are met.

Participants, medical and nursing staff, and investigators (study field staff) will also be blinded to allocation for the probiotic or placebo. The study code will be kept by the study statistician who will have no clinical role and no contact with patients.
After the study's opening assessment is completed (section 5.5.2), the randomisation list will be forwarded to the product distributor (Yakult Europe BV) by the Trial Statistician. Yakult Europe B.V. will arrange for product packs to be delivered, each with a sticker / code specific for each participant. This will ensure the hospital researchers are blinded to verum or placebo.
The placebo will be in identical packaging and of similar appearance and taste. The placebo has been used in several European studies and proven to be effective for blinding purposes.

Study field staff will be unaware to which group the subjects are assigned. See Appendix 5 for the decision tree outlining recruitment and randomisation of subjects. The study code will be kept by the study statistician who will have no clinical role and no contact with patients. Communications via electronic means will be encrypted (AES256), as per Instauration's Standard Operating Procedure.

5.5.4 Product Delivery

Intervention samples will be sent in 15-bottle packs, with a sticker on the front with:

- the name of the PI
- 'Yakult/placebo'
- Patient code
- Best before date

A full set of instructions and dates will be sent. Deliveries (sufficient for two weeks supply per patient) will be every other week on a Tuesday. Orders should be made to Chiyuki Kajita (CKajita@yakulteurope.com), copying Karin Schaareman (KSchaareman@yakulteurope.com), and Kaori Suzuki (ksuzuki@yakulteurope.com). The deadline for ordering is the Wednesday of the week before, six days before the delivery date.
The deliveries will always include a few spare bottles for checking the temperature on receipt. If the temperature is above 7°C, further bottles should be checked. If the whole consignment is at too high a temperature, do not give them to the subjects but inform the above people immediately. They will arrange a second delivery (arriving Wednesday, the next day). This ensures the supply to the patient should not be interrupted. Each site will ensure the fridge is locked and accessed only by study personnel.

5.5.5 Administration of intervention

Participants will receive the study drink within 48 hours of their first dose of antibiotics therapy. After that, participants will receive the verum (probiotic LcS) or placebo drink (65 ml bottle) once a day, according to the randomisation schedule. The study drink will be given by a nurse at drug round.

A schematic representation of the study phases & timeline is presented in *Study flow chart* (Appendix 1 and 2)

Compliance will be monitored throughout the study. As this trial involves inpatients who have proved very compliant in the previous study (Wong *et al* 2014), compliance is defined as follows:

Minor non-compliance: 2 consecutive days not drinking the study intervention;

Major non-compliance: 3 consecutive days not drinking
If a patient misses intervention for more than 3 days, they will be withdrawn from the study.

We will analyse the data using intention-to-treat principle.

An episode of diarrhoea is defined as two or more liquid stools (Bristol Stool Scale types 5, 6 and 7) that is greater than normal for the inpatient over a 24 hour period.
The frequency and duration of diarrhoea / constipation will be monitored.

Patients' quality of life will be assessed by WHO-QoL BREF questionnaire. Furthermore, medication charts (especially antibiotics, laxatives, anti-diarrhoeal agents, proton pump inhibitors and H_2 blocker) and nutrition risk scores (Wong *et al*. 2010, 2012) will be assessed at baseline, 7 days after finishing course of antibiotics and 30 days after finishing course of antibiotics (follow up).

Duration of hospital stay will be recorded on the patients' hospital record.

Bowel function will also be monitored throughout (i.e. stool type; duration of diarrhoea / constipation relapse) by health care staff.

In addition, faecal samples will be routinely acquired at **baseline (within 24 hours of starting intervention, Yakult/placebo), 7 +/- 3 days after finishing course of antibiotics, at follow up (30 +/- 3 days after finishing course of antibiotics), and in the event of diarrhoea.** Study samples will only be collected and processed Mon-Fri, therefore a window period of +/- 3 days for the day 7 and day 30 post-antibiotics course. These samples will be frozen and stored, before sending to YHER for microbiological analysis, including TaqMan PCR analysis of toxigenic *C. difficile.* Technique will be based on the paper by Kubota *et al.* (2014).

Faecal samples from any patient developing diarrhoea will be tested by the hospital according to normal practice and a further sample stored for further analysis by the external laboratory (YHER).

5.5.6 End of Intervention; Start of Follow-up

An episode of diarrhoea will be defined as two or more liquid stools (Bristol Stool Scale types 5, 6 and 7) that is greater than normal for the inpatient over a 24 hour period.
The frequency and duration of diarrhoea / constipation will be monitored.

Patients' quality of life (WHO-QoL: *Spinal Cord* 48:762-769; *Spinal Cord* 50:112-118), medication charts (especially antibiotics, laxatives, anti-diarrhoeal agents, proton pump inhibitors and H_2 blocker) and nutrition risk scores (Wong *et al*., 2012) will be assessed **at baseline (within 24 hours of starting intervention, Yakult/placebo), 7 +/- 3 days after finishing course of antibiotics and at follow up (30 +/- 3 days after finishing course of antibiotics)**.

Duration of hospital stay will be recorded on the patient's hospital record.

Bowel function will also be monitored throughout (i.e. stool type; duration of diarrhoea / constipation relapse).

In addition, faecal samples will be routinely acquired at in the event of diarrhoea, and at the end of intervention. These samples will be frozen and stored, before sending to YHER for microbiological analysis, including TaqMan PCR analysis of toxigenic *C. difficile.* Technique will be based on the paper by Kubota *et al.* (2014).

5.5.7 Study endpoint / closing assessment

An episode of diarrhoea will be defined as two or more liquid stools (Bristol Stool Scale types 5, 6 and 7) that is greater than normal for the inpatient over a 24 hour period. The frequency and duration of diarrhoea / constipation will be monitored.

Patients' quality of life (WHO-QoL: *Spinal Cord* 48:762-769; *Spinal Cord* 50:112-118), medication charts (especially antibiotics, laxatives, anti-diarrhoeal agents, proton pump inhibitors and H_2 blocker) and nutrition risk scores (Wong et la., 2012) will be assessed **at baseline (within 24 hours of starting intervention, Yakult/placebo), 7 +/- 3 days after**

finishing course of antibiotics and **at follow up (30 +/- 3 days after finishing course of antibiotics)**. Duration of hospital stay will be recorded on the patients' hospital record.

Bowel function will also be monitored throughout (i.e. stool type; duration of diarrhoea / constipation relapse).

In addition, faecal samples will be routinely acquired at the end of intervention (7 **+/- 3** days after antibiotics course). These samples will be frozen and stored, before sending to YHER for microbiological analysis, including TaqMan PCR analysis of toxigenic *C. difficile.* Technique will be based on the paper by Kubota *et al.* (2014).

In order to comply with Ethical requirements, Yakult UK would deliver two weeks of Yakult Light to all subjects when they finish the trial. All patients taking Yakult and not in the trial, should be given Yakult Light (blue top) to avoid any muddle with those in the trial who will be drinking red top bottles.

5.5.8 Logistics Stool Specimen

In case participants develop diarrhoea, stool samples will be collected and sent to the trust microbiology laboratory for *Clostridium difficile* toxins analysis.

Collection: Ward nurses will be responsible for collecting stool specimens at baseline and at follow-up for non-routine analysis (SARSTEDT tubes). (Appendix 2)

Transport: Collected stool specimen will be delivered to Buckinghamshire Healthcare NHS Trust's laboratory by hospital porter [equivalent in other SCI centre's site]

Preparation for testing: Baseline and follow up specimen will be collected in SARSTEDT tubes. Diarrhoeal stool specimen x1 will be transfer into 3 specimen pot for further analysis (1x *Clostridium difficile* toxin analysis (in NHS laboratory), 1x PCR/rt-PCR analysis (at YHER), 1x reserve)

Analysis:

- *Clostridium difficile* toxin test will be carried out in BHT's microbiology laboratory under supervision of Dr. Jean O'Driscoll (Consultant Microbiologist)
- Diarrhoeal samples that fall within the trial definition will be tested by the NHS laboratory, and extra samples will be collected: x 1 will be collected for PCR / rt-PCR analysis by YHER, and 1 x reserve (stored).
- If patients develops diarrhoea within 72 hours admission to the SCI centre, then the microbiology department will look for other pathogens that may have been acquired in the community (e.g. *Salmonella, Shigella, Campylobacter*)
- The remainder of the stool specimen will be stored in a dedicated fridge (4 ˚C) for up to 12 hours before being stored at -20 ˚C;
- A spoonful of the stool sample (approximately 200 mg) will be placed in a tube (SARSTEDT, Cat. No. 80.734.001) with 2 ml of RNA*later*

(http://www.sarstedt.com/pdf/prospekte/en/537.pdf). These samples can be frozen at -20 ˚C for 6 months, for delivery to Yakult Europe laboratory for TaqMan PCR analysis of toxigenic *C. difficile* according to the manufacturer's instructions with technique described previously (Kubota *et al.* 2014).

Transport of stool specimen to YHER

Citysprint couriers will be used to courier samples to YHER. Contact details: Victoria Luke (Clinical Trials Co-Ordinator, Tel: 0844 888 4115). Samples will not have any patient identifiable information (with a study project number).

The PI will have a date code and/or additional code to indicate the different samples as follows:

Routine sample #1 (baseline) = collected at baseline within 24 hours of starting intervention (Yakult/placebo). The patient will only just have started antibiotics and intervention (i.e. Yakult or placebo). **Data code: 2 X X X**

Routine sample #2 (ABx+7d) = collected seven days after the patient stops antibiotics (with a window of ±3 days), and thus on the day they stop intervention (Yakult/placebo). **Data code: 3 X X X**

Routine sample #3 (ABx+30d) = collected 30 days after the patients stops antibiotics (with a window of ±3 days), and thus 23±3 days after they stop intervention (Yakult/placebo). **Data code: 4 X X X**

Analysis plan

First YHER will test all **samples#3 (ABx+30d)**. If toxigenic *C. difficile* is found at a prevalence of ≥ 2%, then YHER will test **samples#1 (baseline).**

If a subject develops diarrhoea, the above routine micro testing will be done at Stoke Mandeville. Part of the diarrhoea sample will also be stored in a tube a frozen, before sending for further analysis by YHER. YHER will analyse all diarrhoea samples using TaqMan PCR analysis. (Diarrhoea sample will be code as **5 X X X**)

The decision on whether to test **samples#2 (ABx+7d**) will be taken later.

Samples will be kept at YHER for further microbiota analysis at a later date. This is written into the protocol, and will be highlighted in the informed consent form where patients will be reassured that the samples (and results) will be anonymous.

Analysed data will be sent back to Principal Investigator for decoding (patient's identity).

6. Definitions of outcomes and study variables

6.1 Primary outcome: Occurrence of antibiotic-associated diarrhoea (AAD)

In the current study, occurrence of antibiotic-associated diarrhoea is defined as two or more loose stools (Bristol Stool Scale type 5 or above) up to 30 days after finishing antibiotics.

6.2 Secondary outcomes

6.2.1 Incidence of *C. difficile* diarrhoea (CDAD)

Incidence of *C. difficile* diarrhoea, as diagnosed by a positive *C. difficile* toxin test and stool culture on all patients with undiagnosed diarrhoea. These tests to be conducted by the hospital laboratories.

Bowel function will also be monitored throughout (i.e. stool type; duration of diarrhoea; occurrence of diarrhoea / constipation)

6.2.2 Duration of diarrhoea

In the current study the duration of diarrhoea, defined as two or more loose stools, Bristol Stool Scale type 5 or above, will be collected in addition to the sole occurrence of diarrhoea.

6.2.3 Change in prevalence of *C. difficile* toxin genes

YHER will use TaqMan PCR as a more sensitive test for toxigenic *Clostridium difficile*. This may be done for a subset of samples or all samples, depending on resources available.

6.2.4 Change in gut microbiota (stool specimen analysis)

Further analysis of major bacterial groups in faecal samples may also be performed by YHER.

6.2.5 Quality of life

Patients' quality of life (WHO-QoL: Spinal Cord 48:762-769; Spinal Cord 50:112-118).

6.3 Outcome assessors

Assessments will be carried out by trained, dedicated Research Assistants under supervision of the PI.

7 Statistical planning

7.1 Hypothesis

It is hypothesised that maintenance of a healthy commensal microbiota during antibiotic treatment by administration of a daily probiotic (*Lactobacillus casei* Shirota, as Yakult) will significantly reduce the occurrence of AAD and CDAD, and thus improve quality of life in spinal cord injury patients over time in comparison to the placebo control

The study success is defined as follows: a 17.9% lower occurrence of diarrhoea in the intervention group when compared to the control group.

7.2 Sample size considerations

Based on pilot data, this study aimed to find a difference of 17.9% in the proportion of patients with diarrhoea in two groups, (i) those on proton pump inhibitors – 56.3% and (ii) those on LcS – 38.4%. With α=0.05 and power of 90%, we estimate a sample size of 162 per group. After accounting for dropouts at 10% (across the length of the trial) this will require 180 per arm (total n=360).

This sample size calculation is based on data from a single study site, but it seems reasonable to extrapolate the findings to other UK SCI centres. Therefore we aim to recruit 360 (180 per arm) in the proposed trial.

7.3 Sample per Investigative Site

Estimated annual recruitment rates for the following participating centres are:

- National Spinal Injuries Centre, Stoke Mandeville Hospital: 9/month; 108 /year.
- The Midlands Centre for Spinal Injury, The RJ&AHO Hospital: 4/month; 48 /year.
- The Princess Royal Spinal Injuries Centre, Northern General Hospital: 3/month; 36/year.

7.4 Statistical analysis

We will analyse the data using intention-to-treat principle. Fisher's exact test and χ2 test will be used to compare rates of diarrhoea, as well as rates of AAD and CDAD. Relative risk and the number needed to treat, both with 95% confidence intervals, will be used to describe the treatment effect of probiotic. Logistic regression will be used to establish which factors influence the occurrence of diarrhoea. Continuous outcome measures will be analysed utilising ANCOVA (controlling for baseline scores and existing group differences) and multiple regression analyses ($p<0.05$ critical value; sample size covers this analysis to a sufficient power level).

To reduce the bias implicit in utilising only complete cases, multiple imputation procedures for the laboratory data will be used using the SPSS (SPSS version 15, Inc, Chicago, IL) Markov Chain Monte Carlo multiple imputation function to produce 10 imputed datasets. These will then be analysed as normal; thereafter standard multiple

imputation procedures were used to combine the multiple scalar and multivariate estimates quantities.

To minimise confounding factors with respect to incidence of AAD and CDAD, stratification will be performed at the time of recruitment according to the antibiotic background (i) patients who have not previously received antibiotics in the 30 days prior to administration of intervention (‘antibiotic-naïve’) and (ii) patients who had been given a single antibiotic treatment for prophylactic purpose >14 days before first administration of intervention (‘prophylactic-antibiotic’).

In addition, a sub-group analysis will be performed on both treatment arms to analyse the following for association with AAD/CDAD occurrence: (i) risk of undernutrition, (ii) use of proton pump inhibitor, (iii) age ≥ 65 years and (iv) those receiving study drink within 24 hrs vs 48 hrs of first dose of antibiotics.

7.5 Interim Analysis

Interim (safety) analysis is set to be conducted when recruitment levels reach 30% of the total calculated sample size, or 12 months after the start of data collection, whichever occurs sooner. This will not involve unblinding of the data, unless there are serious concerns that adverse events are unexpectedly high.

In addition the funding provider will appoint an external consultant to monitor this trial at both sites. It is anticipated that the schedule of monitoring will be

a. An initiation visit to each site
b. Two interim visits to centres 1 and 2
   1: after 50 patients are recruited at site 1 and 20 recruited at site 2
   2: after 150 patients recruited at site 1 and 60 recruited at site 2
c. One interim visit to centre 3 after 20 patients are recruited
d. Close out visit / site after full recruitment at each site

8. Patient risk analysis

8.1 Risks

We do not expect any risk in this study as the high risk individuals will be excluded from the study. The probiotic drink we use is a commercially available product; it has been used in trials in UK and Europe since mid-1990s, with no reported safety issues. (Hammerman C. *et al*. (2006) Safety of probiotics: comparison of two popular strains. BMJ 333, 1006-8.) There are numerous reports of safe use of this probiotic strain in a wide range of patients, some of them seriously ill.

The use of LcS was also shown as safe for use in acute SCI patients in our previous trial (Wong S et al, 2014).

The occurrence of adverse event (such as diarrhoea) is expected to be high in the subject group (Wong *et al*., 2014 open label study paper) but incidence of diarrhoea is the primary outcome of the study.

The probiotic used in the current trial has been reviewed by the Buckinghamshire Healthcare NHS Trust Department of Microbiology, Director of Academic Pharmacy and Associate Chief Nurse and approved for use, subject to Ethics approval.

Buckinghamshire Healthcare NHS Trust indemnity scheme will apply for potential legal liability of sponsor (s) or employer(s) for harm to participants arising from the design of the research.

8.2 Benefits

There will be no benefits for the study subjects in this study, except for the close study-specific monitoring of their physical and mental status.

9 Adverse Event reporting

9.1 Adverse Events

Adverse events are defined as any unfavourable and unintended diagnosis, symptom, sign (including an abnormal laboratory finding), syndrome or disease which either occurs during the study, having been absent at baseline, or, if present at baseline, appears to worsen. All adverse events occurring during the study must be recorded in the patient's Case Report Form (CRF).

9.2 Serious Adverse Events (SAE)

Serious AE's (SAE's) are defined as any untoward medical occurrences that: (1) result in death, (2) are life threatening, (3) require (or prolong) hospitalization,(4) cause persistent or significant disability/incapacity, (5) result in congenital anomalies or birth defects, or (6) are other conditions which in the judgment of the investigators represent significant hazards.

An adverse event is considered life threatening if, in the view of the investigator, the participant was at immediate risk of death from the event as it occurred. An adverse event is considered incapacitating or disabling if the experience results in a substantial and/or permanent disruption of the subject's ability to carry out normal life functions. Any AE that because of its seriousness requires hospitalisation represents an SAE.

Because of the need to report to the Institutional Review Board all serious adverse events in a timely manner, it is vitally important that an investigator report any SAEs immediately, even if the investigator does not consider the adverse event to be product-related. SAEs must be documented. In addition, SAEs must be e-mailed or faxed to the sponsor's clinical research manager, the principal investigator,the local IRB and the financial sponsor, independent of causality within 24 hours so that reporting requirements to regulatory authorities can be met within the required time frame. Any SAE which occurs

during the study, whether or not related to the study must at all times be reported. We will only collect unexpected AEs (outside of normal clinical presentation for SCI patients or any worsening of a baseline condition) and SAEs (expected or unexpected). All unexpected SAEs will be emailed or faxed to the local IRB within 24 hours so that reporting requirements to regulatory authorities can be met. For expected SAEs we will report to IRB, study sponsor (Institution's R&D) and financial sponsor (Yakult) at regular intervals. Study monitor will be copied in for monitoring purpose.

If an AE is judged to be serious and related to the study treatment, the treatment must be terminated immediately or as quickly as possible without risking harm to the patient. If an AE is judged to be mild, moderate, or severe, the decision about whether or not to stop the study (temporarily or permanently) must be made.

9.3 Product-related anticipated adverse events

As outlined under *heading 8.1*, no adverse events other than observed in regular clinical neurorehabilitation are expected to occur during the study.

9.4 Adverse event reporting

For each AE and SAE the following information will be collected:

- Name and description of the event
- Date of the start of the event (if applicable)
- Actions taken due to the occurrence of the event
- Outcome of the event
- Date the patient has recovered from the event
- Relationship to product or treatment under investigation
- Severity of the event
- Seriousness of the event

If detailed information regarding an AE is not available at the time of initial reporting, completion of the AE form must be achieved at the next possible time point.

As soon as a study staff member becomes aware of an SAE, this person will ensure that the following people are notified:

- Principal Coordinating Investigator (PCI)
- Sponsor: For regulated studies (e.g. investigational product exemption), the sponsor will also report this information to the appropriate regulatory body.Ethics Committee (EC) / Institutional Review Board (IRB)
- Financial Sponsor

10 Data management

10.1 Data collection, source data, storage and archiving

All paper documents pertaining to the study will not leave the hospital and will be stored as per local policy. For this study, a Case Report Form (CRF) is designed containing data items as specified in this CRP. Modification of the CRF will be made only if deemed necessary and in accordance with any amendment to the CIP. It is the study coordinator's, or designated person's, responsibility to complete the CRF during the conduct of the study.
CRFs will be used to collect all subject data during the study. CRFs must be fully completed for each subject, signed, and available for review. The CRF must be a controlled document that ensures numbering and patient's anonymity. For every volunteer who reached the endpoint of the study, the PI must review and sign the CRFs including for those removed from the study for any reason. After the study closure, the original copy along with all other support documents will be retained by the investigational site. The investigator will retain a copy of the completed signed case report forms.
The investigators file should be archived in agreement with national laws. The investigator file should be kept for 15 years. The sponsor should be informed where the investigator file is stored and archived. The investigator file includes the following documents:

Patient Documents.

- Signed Informed Consent
- Study protocol and amendments, including training modules
- Product user's Guide
- Screening form
- Case Report Form (CRF)
- Adverse Event form
- List of the study personnel involved in the study with their signatures
- The curricula vitae of the PCI and other members
- Study / monitoring reports

Investigator's Documents

- Investigator's contract with information on tasks and responsibilities and adjustments.
- Financial contract

Approvals

- Approval from the IRB / Ethical Committee for the study protocol and for major amendments of the protocol
- Notification to the authorities
- Other correspondence with committees and regulatory bodies

10.2 Confidentiality

Privacy and confidentiality of the patient's medical data will be maintained through the study. CRFs and all other documents sent to the Sponsor will be de-identified and carry

only the numeric patient's identifier code. The sites will maintain the link between the patient identifier code and the patient's names.
Fully identifiable information may be reviewed for the purpose of verifying data in the CRFs and only at the location of the investigational sites. This can be carried out by the Sponsor or Sponsor's designee, regulatory agencies or authorised quality assurance personnel. Investigation clinic specific regulations and procedures may apply and will be followed by the above listed personnel. Personal medical information will at all times be treated as confidential. The informed consent document will contain information about the confidentiality of the medical information and approval for the access.

All confidentially marked documentation and data related to this study will be stored in locked file cabinets in secure areas under lock and key; such data may not be left unattended at any time, nor may it be stored in locations other than the specified cabinet.
No patient identifiers will be entered in the electronic database, all patients will be identified as their study number only. The database will be stored on a private network at Buckinghamshire Healthcare NHS Trust or The Robert Jones & Agnes Hunt Orthopaedic Hospital (depending on site of recruitment), only accessible through specifically created portals. Access to the electronic database will be based on a unique username and password. The password will be a minimum of 6 characters long with combination of character and numbers. Password may be changed by the user after account creation if desired. Password fields will always be non-readable (character replaced with dots). A separate document containing the key linking patient identifiers and study numbers will be kept at the investigative site's premises at a secure place, only accessible by the local investigative team.

11 Study Management and Quality Control

The study will be monitored by an external Clinical Research Associate according to applicable provisions of the sponsor's procedures or sponsor's subcontractors monitoring procedures, in conformance with ICH-GCP FDA guidelines, ISO 14155 and specific country laws/regulations. Accordingly, monitoring visits to the investigative site shall be made periodically during the feasibility study, to ensure that all aspects of the current approved protocol and amendment(s) are followed.

12 Regulatory aspects

This trial shall be performed in accordance with the ICH-GCP, FDA Guidelines, World Medical Association Declaration of Helsinki, and will follow the guidelines for conducting a clinical investigation in Europe as outlined in the European Standard EN ISO 14155.
The clinical protocol must be reviewed and approved by the participating centres' IRBs and the National Research Ethics Committee before subject enrolment may begin. All proposed changes to the clinical protocol will be declared, sent out for review and approval in writing to the Research Ethics Committee. These changes will be considered as Protocol Amendments. All changes will be consistent with abovementioned

regulations. The PI and Financial Sponsor's representative will both sign the protocol and any amendments prior to implementing it at participating centres.

13 Study report and publication policy

13.1 Final report

The results of the statistical evaluation will be summarised in a report, which forms the basis for the comprehensive final report. The comprehensive final report forms the basis for all future publications. The PI will be in charge of coordinating the development of the final report. The final report will be drafted by the PI and co-investigators.

13.2 Publication & Presentation

The publication strategy will be decided between the PI and co-investigators and any other affected party (in End of study meeting). Subject to scientific merit and contribution, a publication and conference presentation strategy will be agreed upon by the PI and co-investigators.

The study sponsor and the financial sponsor is aware of, but did not influence the trial design, and had / will have no role in the data analysis and interpretation.

14 Termination criteria

14.1 Premature Termination of the Procedure

The planned study may be prematurely terminated for a variety of reasons including product faults or failures and significant health complications or desire expressed by the patient not to continue with the trial.

A product fault is an unexpected change to the product, or placebo, contradictory to the instructions for use and may or may not affect product performance. All product faults will be documented on the CRF for analysis and will be communicated with both the product manufacturer/distributer and Sponsor.

14.2 Removal of Patients from the study

Patients will be removed from the study whenever it is necessary to safeguard their health and welfare. Non-compliance with the protocol or occurrence of significant adverse events may also necessitate discontinuation from the study. When a patient is permanently removed from the study, a study closing assessment should be performed if possible, and all results must be obtained. Patients removed from the study because of an adverse event will be followed-up until the adverse event has resolved.

14.3 Termination of the study

The progress of the study, in particular the enrolment and safety aspects, will be closely monitored together with the Sponsor and the financial sponsor. If the predicted enrolment is not met and the study is not likely to progress as planned, the sponsor (together with the PI and co-PIs and the financial sponsor) may decide to terminate the study. In the case of an early termination of the study, all patients already enrolled in the study will be followed up until the last FU visit as defined in the CRP. The Research Ethics Committee will be informed about developments related to study termination.

15 Time schedule

This study will be conducted over a period of approximately 46 months. The first 43 months will be allocated to recruitment, follow-up and prospective data collection, the final 3 months will be used for data analysis and report writing.

Time	Event	Status
Nov 12 – Feb 12	Full manuscript (Part I study)	Submitted on 4th April 2013
April 2013	Consultation with Health Service Research Unit, City University London	Trial Statistician identified
May 2013	Submission to NSIC Research Board	Approved
Nov 2013	Anticipate decision from Yakult regarding product / placebo provision and microbiota analysis	Approved
19 December 2013	Pre-study meetings with Yakult Europe / Yakult UK	Approved
30 July 2014	Contract agreement signed with study financial sponsor (YHL)	Approved
	Ethics Application (anticipate this will take 3 months)	Approved
	Recruitment:<br>Approximately 13 patients per month [Centre 1: 7 per month; Centre 2: 3 per month; Centre 3: 3 per month]<br>Total recruitment period: 43 months<br>Aim to complete follow up data: month 44	
	Interim meetings (Interim safety analysis)	
	Data analysis	

	Report writing	

16 Finances

Attached in a separate document.

17 Appendices

Appendix 1: Study flow chart

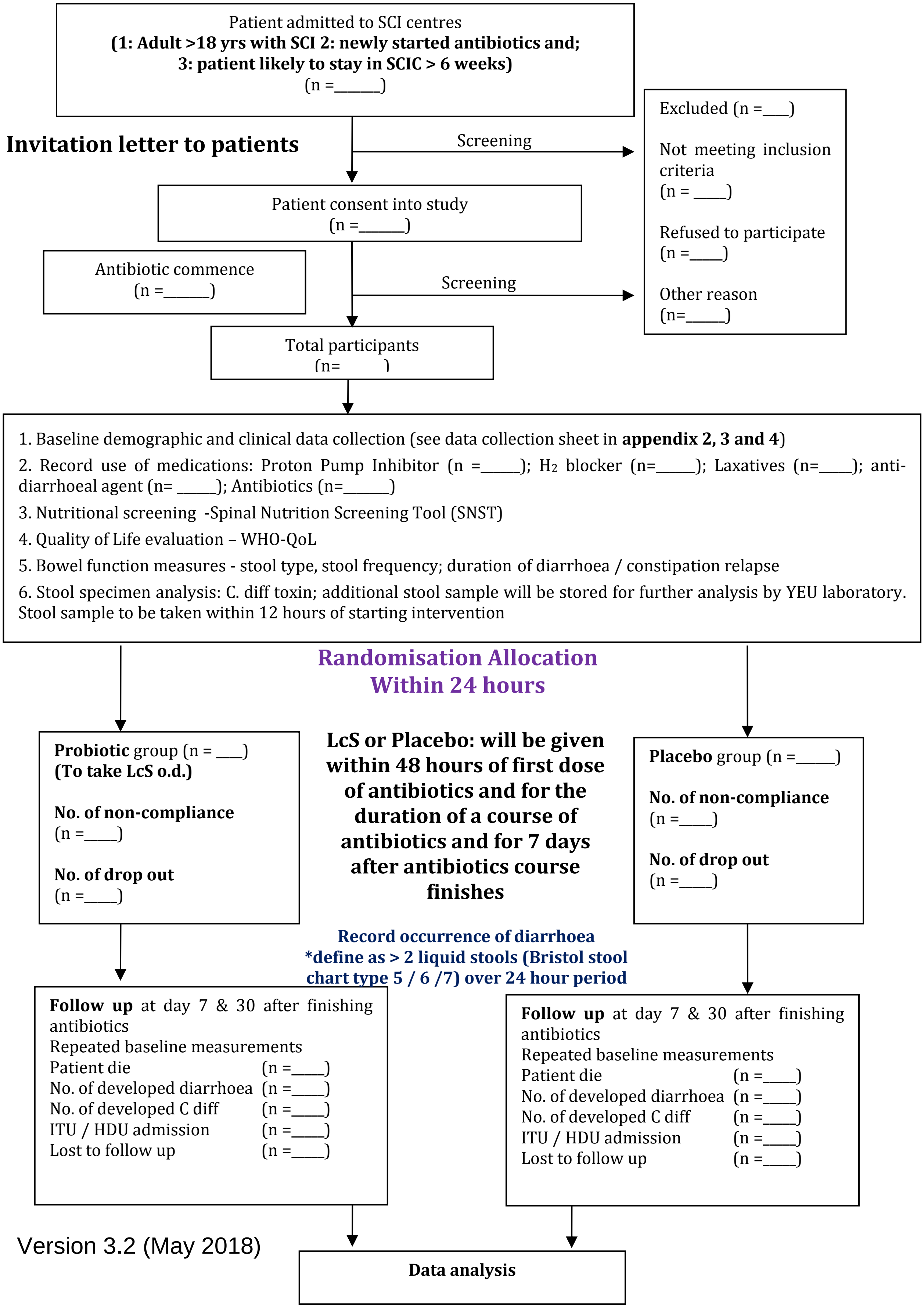

**Appendix 2. Study data collection – summary**	**Screening / Opening visit**	**(Probiotic / Placebo allocation)**	**Monitor Daily (+) weekly (x) throughout the study**	**7 days after finishing antibiotics course**	**30 days after finishing antibiotics course**
**Eligibility**	X				
**Informed consent**	X				
**Demographics & Clinical data**	X			X	X
**Compliance**			+	X	
**Record use of medications**	X				
Proton pump inhibitor				X	X
H_2 blocker	X			X	X
Anti-diarrhoeal agent	X			X	X
Antibiotics	X		+	X	X
**Nutrition risk screening- SNST**	X			X	X
**Quality of life – WHO-QoL**	X			X +/- 3 days	X +/- 3 days
**Bowel function assessment**					
Stool type (Bristol scale)	X		+	X	X
Occurrence of diarrhoea			+	X	X
Occurrence of constipation			+	X	X
Stool specimen collection	X		*	X +/- 3 days	X +/- 3 days
C. difficile toxin analysis	X		*	x	X
Other Pathogens	X		*	x	X

***IF diarrhoea develops, additional diarrhoea sample will collected and stored for further analysis by YHER**

Version 3.2 (May 2018)

Appendix 3: Study Gantt Chart

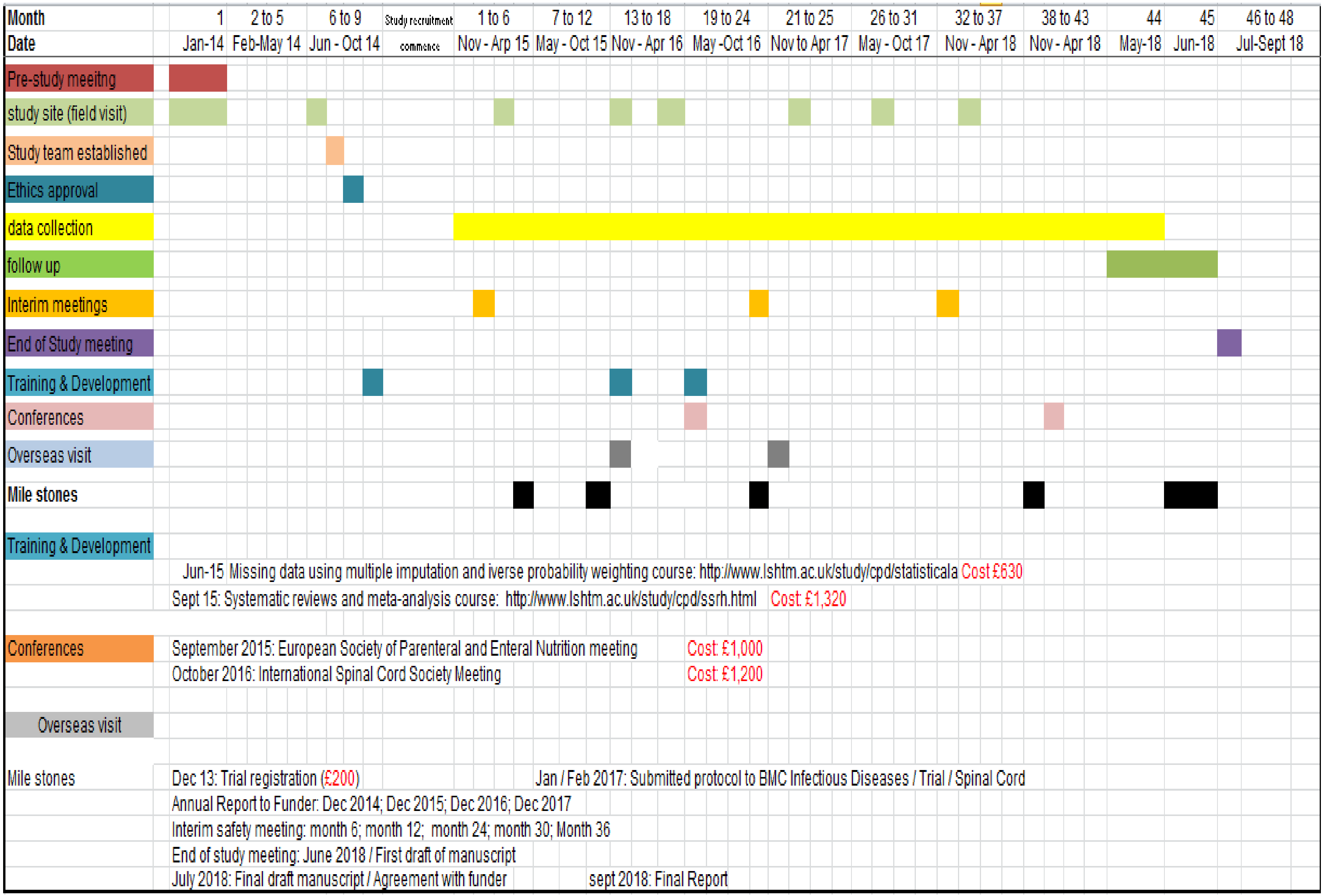

Appendix 4: CONSORT 2010 checklist of information to include when reporting a randomised trial*

Section/Topic	Item No	Checklist item	Reported On page no
**Title and abstract**			
	1a	Identification as a randomised trial in the title	1
	1b	Structured summary of trial design, methods, results, and conclusions (for specific guidance see CONSORT for abstracts)	4
**Introduction**			
Background and objectives	2a	Scientific background and explanation of rationale	10
	2b	Specific objectives or hypotheses	11
**Methods**			
Trial design	3a	Description of trial design (such as parallel, factorial) including allocation ratio	11
	3b	Important changes to methods after trial commencement (such as eligibility criteria), with reasons	/
Participants	4a	Eligibility criteria for participants	11
	4b	Settings and locations where the data were collected	9
Interventions	5	The interventions for each group with sufficient details to allow replication, including how and when they were actually administered	12, 14, 15
Outcomes	6a	Completely defined pre-specified primary and secondary outcome measures, including how and when they were assessed	19
	6b	Any changes to trial outcomes after the trial commenced, with reasons	/
Sample size	7a	How sample size was determined	20
	7b	When applicable, explanation of any interim analyses and stopping guidelines	21, 26
Randomisation:			14
Sequence generation	8a	Method used to generate the random allocation sequence	14
	8b	Type of randomisation; details of any restriction (such as blocking and block size)	14
Allocation concealment mechanism	9	Mechanism used to implement the random allocation sequence (such as sequentially numbered containers), describing any steps taken to conceal the sequence until interventions were assigned	14
Implementation	10	Who generated the random allocation sequence, who enrolled participants, and who assigned participants to interventions	14
Blinding	11a	If done, who was blinded after assignment to interventions (for example, participants, care providers, those assessing outcomes) and how	14

	11b	If relevant, description of the similarity of interventions	14
Statistical methods	12a	Statistical methods used to compare groups for primary and secondary outcomes	/
	12b	Methods for additional analyses, such as subgroup analyses and adjusted analyses	21
**Results**			
Participant flow (a diagram is strongly recommended)	13a	For each group, the numbers of participants who were randomly assigned, received intended treatment, and were analysed for the primary outcome	28
	13b	For each group, losses and exclusions after randomisation, together with reasons	15, 28
Recruitment	14a	Dates defining the periods of recruitment and follow-up	15, 16
	14b	Why the trial ended or was stopped	/
Baseline data	15	A table showing baseline demographic and clinical characteristics for each group	/
Numbers analysed	16	For each group, number of participants (denominator) included in each analysis and whether the analysis was by original assigned groups	/
Outcomes and estimation	17a	For each primary and secondary outcome, results for each group, and the estimated effect size and its precision (such as 95% confidence interval)	/
	17b	For binary outcomes, presentation of both absolute and relative effect sizes is recommended	/
Ancillary analyses	18	Results of any other analyses performed, including subgroup analyses and adjusted analyses, distinguishing pre-specified from exploratory	/
Harms	19	All important harms or unintended effects in each group (for specific guidance see CONSORT for harms)	/
**Discussion**			
Limitations	20	Trial limitations, addressing sources of potential bias, imprecision, and, if relevant, multiplicity of analyses	/
Generalisability	21	Generalisability (external validity, applicability) of the trial findings	/
Interpretation	22	Interpretation consistent with results, balancing benefits and harms, and considering other relevant evidence	/
**Other information**			
Registration	23	Registration number and name of trial registry	4
Protocol	24	Where the full trial protocol can be accessed, if available	BMC journals
Funding	25	Sources of funding and other support (such as supply of drugs), role of funders	4

*We strongly recommend reading this statement in conjunction with the CONSORT 2010 Explanation and Elaboration for important clarifications on all the items. If relevant, we also recommend reading CONSORT extensions for cluster randomised trials, non-inferiority and equivalence trials, non-pharmacological treatments, herbal interventions, and pragmatic trials. Additional extensions are forthcoming: for those and for up to date references relevant to this checklist, see www.consort-statement.org.

Appendix 5. Decision tree for recruitment and randomisation

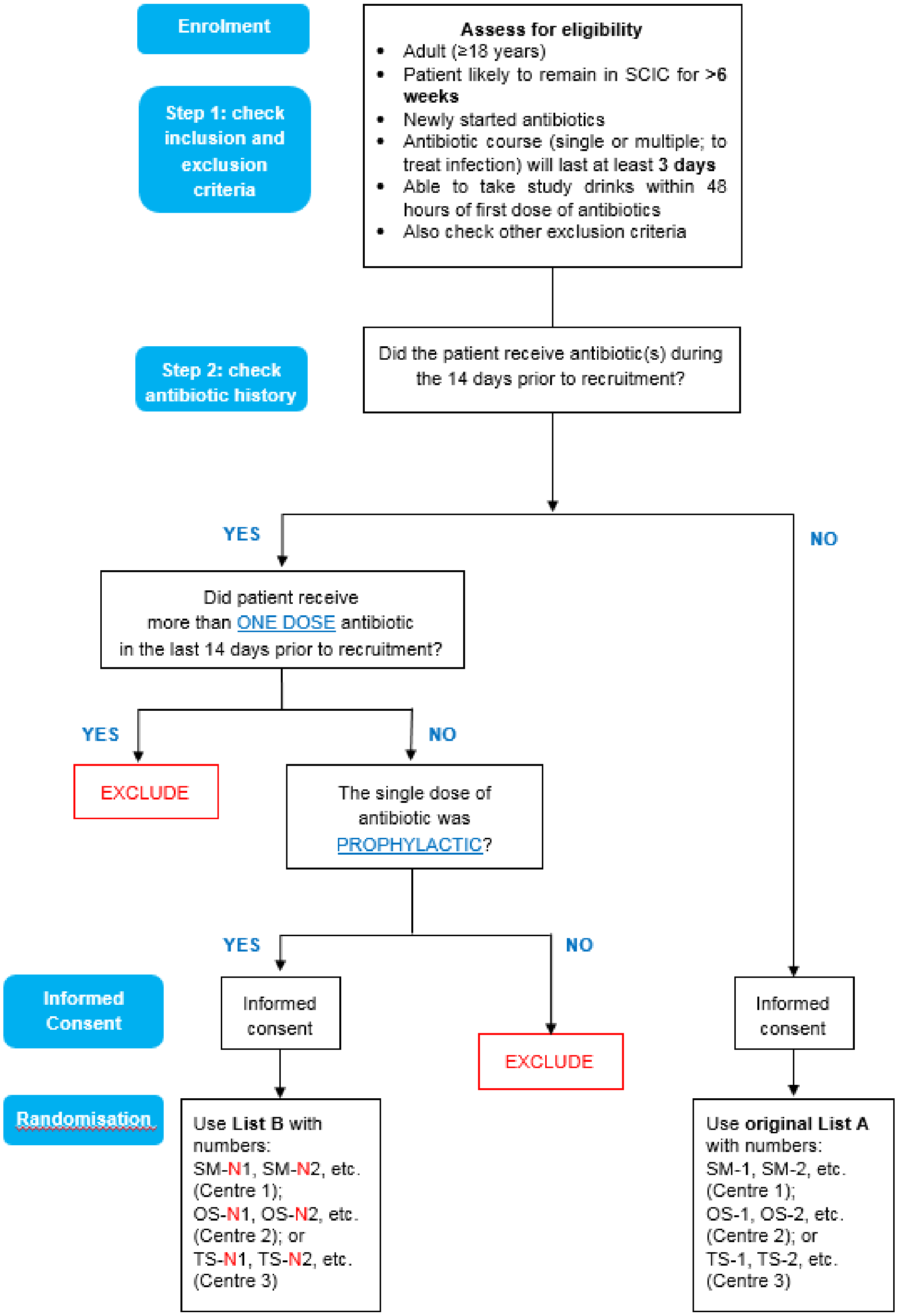

18 References

Consortium for Spinal Cord Medicine**.** *Early Acute Management in Adults with Spinal Cord Injury: A Clinical Practice Guideline for Health-Care Providers*. Paralyzed Veterans of America, Washington DC, 2008.

Drossman DA*.* Rome III: the new criteria. *Chin J Dig Dis.* 2006;7(4):181-5.

Joint Food and Agriculture Organization of the United Nations (FAO) and the World Health Organization (WHO). Report of a Joint FAO / WHO Working Group on Drafting Guidelines for the Evaluation of Probiotics in Food. London, Ontario, Canada, April 30 and May 1, 2002.

Health Protection Agency / Department of Health. *Clostridium difficile* infection: How to deal with the problem. Department of Health, London, 2008.

Kubota H, Sakai T, Gawad A, *et al*. Development of TaqMan-Based Quantitative PCR for Sensitive and Selective Detection of Toxigenic *Clostridium difficile* in Human Stools. PLoS ONE. 2014;9(10): e111684. doi:10.1371/journal.pone.0111684.

Lewis JN, Thomas LV and Weir W (2009). The potential of probiotic fermented milk products in reducing risk of antibiotic-associated diarrhoea and *Clostridium difficile* disease. *Int J Dairy Technol* **62(4)**:461-471.

Martinez CO, Paz VS and O'Donnell A. *Lactobacillus casei* Shirota prevention approach for healthy children with mild acute infection treated with antibiotics. *Pediatric Research* 2003;**4(2)S,** 174A.

O'Donnell LD, Virjee J, Heaton KW. Detection of pseudodiarrhoea by simple clinical assessment of intestinal transit time. *Br Med J* 1990;**300**:439-440.

Pirker A, Stockenhuber A, Remely M, *et al.* Effects of antibiotic therapy on the gastrointestinal microbiota and the influence of *Lactobacillus casei*. *Food Agricult Immunol* 2013;24(3):315-330. doi 10.1080/09540105.2012.689816

Wong S, Derry F, Jamous A, *et al.* Validation of the Spinal Nutrition Screening Tool (SNST) in patients with spinal cord injuries (SCI) - result from a multicentre study. *Eur J Clin Nutr* 2012; **66**, 382-387.

Version 3.2 (May 2018)

Wong S, Jamous A, O'Driscoll J, *et al*. A *Lactobacillus casei* Shirota probiotic drink reduces antibiotic-associated diarrhoea in patients with spinal cord injuries: a randomised controlled trial. *Br J Nutr* 2014 Feb;111(4):672-8. doi: 10.1017/S0007114513002973. Epub 2013 Sep 18.
