# Supplementary material for: Lactobacillus casei Shirota probiotic drinks reduce antibiotic associated diarrhoea in patients with spinal cord injuries who regularly consume proton pump inhibitors: a subgroup analysis of the ECLISP multicentre RCT
Source: Spinal Cord. 2024 Mar 22;62(5):255–63. doi: 10.1038/s41393-024-00983-w (PMC11176055; doi:10.1038/s41393-024-00983-w)
Supplement: Supplementary file 2 [file 41393_2024_983_MOESM2_ESM.docx]

Antibiotics risk table for *Clostridium difficile* associated diarrhoea / antibiotic associated diarrhoea

| **No** | **Name** | **Risk** | **n** |
| --- | --- | --- | --- |
| **1** | Gentamicin | Low | 25 |
| **2** | Trimethoprim | Low | 21 |
| **3** | Tazocin | Medium | 16 |
| **4** | Nitrofurantoin | Low | 12 |
| **5** | Flucloxacillin | Low | 11 |
| **6** | Amoxicillin | High | 8 |
| **7** | Teicoplanin | Low | 7 |
| **8** | Ciprofloxcin | High | 6 |
| **9** | Metronidazole | Low | 6 |
| **10** | Pivecillinam | Low | 6 |
| **11** | Clarithromycin | Low | 5 |
| **12** | Vancomycin | Low | 4 |
| **13** | Temocillin | Low | 3 |
| **14** | Doxycycline | Low | 3 |
| **15** | Phenoxymethypenicillin | Low | 2 |
| **16** | Co-Amoxiclav | High | 1 |
| **17** | Meropenem | Medium | 1 |
| **18** | Erythromycin | Low | 1 |
| **19** | Fucidic acid | Low | 1 |

**Eligibility of subjects for participation in the ECLISP study.**

**Inclusion criteria Exclusion criteria**

1. Subject >18 years of age 1. Re-recruit patient

2. Sustained a spinal cord injury 2. Antibiotic for prophylaxis up to 14 days prior to recruitment

3. Likely to remain in-patient > 6 weeks 3. Diarrhoea within the preceding 7 days prior to recruitment

4. Due to receive antibiotic for infection 4. Bowel pathology that could result in diarrhoea

5. On regular proton pump inhibitor 5. Bowel surgery in last 6 months

6. Infective endocarditis

7. Active inflammatory bowel disease

8. Pancreatitis

9. Regular probiotic use in the previous 8 weeks

10. Antibiotic use in the 30 days before intervention

11. Immunosuppression

12. Nil by mouth / non-functioning gut

13. Known cow’s milk protein intolerance

14. Psychiatric / cognitive conditions that may interfere the study

15. Those unable to give informed consent due to cognitive impairment

16. Pregnant or breastfeeding women

17. Prisoners

| ECLISP protocol amendment history | | | |
| --- | --- | --- | --- |
| Amendment no. | Protocol version | Date issued | Details of change made |
| 1 | 1.13 | 23 June 2014 | Minor amendment: 7^th^ August 2014  Changes are made as we previously reported (all patients =360), instead of “each patient” |
| 2 | 1.13 | 23 June 2014 | Minor amendment: 22^nd^ September 2014  Clarification regarding storage of sample  Letter of invitation to participant [Consultant invitation letter, version 2.0, date 22 Sept 2014)  Participant consent form  [version 2.0; 22^nd^ September 2014]  Participant information sheet  [version 4.0, 22^nd^ September 2014] |
| 3 | 1.13 | 25 Sept 2014 | Minor amendment: 25 September 2014  Grammatical changes to PIS and consent form  Participant consent form [version 3, date: 25 September 2015]  Participant information sheet [ version 5, date: 25 September 2015] |
| 4 | 1.14 | 3 October 2014 | Notice of substantial amendment 1 (21 October 2014)   1. Removed blood biochemistry from the data collection sheet 2. Amended the study protocol to clarify    1. Informed consent procedure; Those not able to give written consent    2. Update exclusion criteria    3. We added +/- 3 days window period for follow-up specimen data collection    4. Procedure for AEs and SAEs.       1. We will collect / report all un-expected SAEs within 24 hours       2. We will collect / report all expected AEs, and SAEs regularly    5. We update the MCSI’s PI name   We will record Major non-compliance as withdraw. We will analysis the data using intention-to-treat principle |
| 5. | 1.15 | 22 May 2015 | Notice of minor / substantial amendment 2 (DATE)  Change in exclusion criteria   - Antibiotics for prophylaxis use in the last 14 days prior to the study product first administration (instead of 30 days) - More than one dose of prophylactic antibiotic given for prophylaxis use administered in the last 14 days prior to commencement of intervention - Antibiotic use in the 30 days prior to the study product first administration (apart from one time prophylactic use more than 14 days prior to commencement, as described above)   Change in contact detail   - Yakult Honsha European Research Centre for Microbiology   Clarification in statistics analysis |
| 6. | 1.16 | 25^th^ June 2015 | Amendment to protocol:-  Exclusion criteria  Change in exclusion criteria   - Antibiotics for prophylaxis use in the last 14 days prior to the study product first administration (instead of 30 days) - More than one dose of prophylactic antibiotic given for prophylaxis use administered in the last 14 days prior to commencement of intervention - Antibiotic use in the 30 days prior to the study product first administration (apart from one time prophylactic use more than 14 days prior to commencement, as described above)   Change in contact detail   - Yakult Honsha European Research Centre for Microbiology   Clarification in statistics analysis |
| 7. | 2.0 | 7^th^ Sept 2015 | Version 2.0 with the update inclusion / exclusion criteria; recruitment and consent procedure), update participant information sheet and consent form.  Inclusion /exclusion criteria:-  Newly started antibiotics (the course of antibiotics used as treatment for an infection should be a minimum of 3 days; single or multiple)  Able to take study drinks within 48 hours of first dose of antibiotics. |
| 8. | 3.1 | 15^th^ May 2017 | This amendment include an update protocol (version 3.1) with the following updates   - New recruiting centre: The Princess Royal Spinal Injuries Unit at Northern General Hospital, Sheffield Teaching Hospitals NHS Foundation Trust. - New local PI and co-investigators for Sheffield’s centre. - Amended study end-date as the recruitment was slower than we expect. - Updated appendix 5: decision tree for recruitment and randomisation - Updated **window period** for baseline and follow up data collection. |
| 9. | 3.2 | 17^th^ May 2018 | Update financial sponsor information  Update email for study investigators  Update study Gannt chart to reflect new study end date. |
